# An original fuzzy control approach for active anti-roll bars to prevent rollover

**DOI:** 10.1371/journal.pone.0290409

**Published:** 2023-11-27

**Authors:** Tuan Anh Nguyen

**Affiliations:** Thuyloi University, Hanoi, Vietnam; University of Hull, UNITED KINGDOM

## Abstract

In this article, the author introduces a new fuzzy control solution to direct active anti-roll bars (hydraulic stabilizer bars) in order to enhance the vehicle’s roll stabilization efficiency. The original fuzzy algorithm designed in this work can satisfy all the roll stability, comfort, and response speed requirements, while previous algorithms could only meet one of these criteria. In addition, a fully dynamic model is established to simulate the vehicle’s roll oscillations instead of only using a simple half-dynamic model combined with the single-track dynamic model. The calculation and simulation processes take place in the Simulink environment. Two cases of steering are used as input to the simulation problem; the car’s speed is gradually increased through three levels. According to results of research, the roll angle and roll index of the car increase as the speed and steering angle increase. The interaction between the road and wheel decreases sharply as the roll angle increases, which can lead to a rollover. In the first case, the rollover occurs only when the car travels at *v*_*3*_ without the stabilizer bar and has a maximum roll angle of 9.81°. In the second case, this occurs for the (None) situation when traveling at speed *v*_*1*_ with a maximum roll angle of 9.52° and for the (Passive) situation when traveling at speed *v*_*2*_ with a peak value of 11.93°. Meanwhile, the vehicle’s stability is still well guaranteed when utilizing active anti-roll bars controlled by an original fuzzy algorithm.

## 1. Introduction

Rollovers are one of the most catastrophic accidents and are more dangerous than other accidents [1]. According to Khan and Vachal, rollover accidents account for only about 1.7% of all crashes in the United States, but the number of deaths related to these crashes can be as high as 33% [2]. These figures for Mexico are 5% and 35%, respectively [2]. In China, 5036 rollover accidents occurred in 2016, which accounted for only 2.68% of total car accidents, but 5.64% of total fatal accidents, which was more than double the amount, according to Wen et al. [3]. According to another report cited in [4], the fatality rate from rollover accidents can be ten times higher than in other accidents (under certain conditions).

There are several causes of rollover accidents, which can be divided into 3 main sections: external, internal, and the driver’s behavior. For external factors, road-tripping is considered the first factor [5]. However, if the height of the trip is low, its effect will not be large. According to Yin et al., slope and road geometry have an important influence on the vehicle’s lateral instability, which is the precursor to rollover [6]. The contact between the wheels and the road is characterized by the ability to hold between them. This interaction is easily lost in bad weather conditions, such as wet or snowy weather, leading to destabilization phenomena [7]. For large vehicles, such as SUVs or trucks, the effect of crosswind on rollover is significant [8, 9]. These are known as external non-parametric uncertainties, and they can be determined using statistical approaches and incorporated into the main model. Designing an adaptive controller can handle these uncertainties. As for the internal factors, the technical parameters of the car also greatly influence the instability of the car when steering. According to Kongwat et al., optimal mass distribution of body positions is necessary to help avoid rollover [10]. The structure of the chassis needs to be properly calculated to help optimize the mass distribution of passengers and cargo [11]. Vehicle dimensions, such as, track width, the height of the center of gravity, etc., play an essential role in deciding when to experience the phenomenon of rolling over [12]. Even the effect of the kingpin angle can be considered if a more accurate assessment of rollover models is desired [13]. Both factors mentioned above only partially affect the phenomenon of vehicle rollover, while the driver’s control behavior plays a decisive role in this issue. In [14], the car’s speed is mentioned as one of the main causes of vehicle roll instability. Besides, the steering angle and angular acceleration also greatly contribute to the rollover situation when traveling at high speed [15]. In actual rollover accidents, the driver’s control is considered the most critical factor in determining whether the vehicle will roll over.


$$RI=|\frac{F_{z1}-F_{z2}}{F_{z1}+F_{z2}}|$$ (1)

Rolling over can be assessed through many criteria, of which the roll index is one of the essential indicators often used in many studies. The roll index (*RI*), is described through the Eq (1). In simple terms, this index is determined based on the difference in a dynamic load between wheels. If the car’s roll angle is not large, this index is rewritten as the Eq (2), according to Tian et al. [16]. The roll index value is 1 when the automobile is completely rolled over. To assess the danger of rollover, Chao et al. divide this index into five corresponding levels [17]. The value of the roll index can be accurately calculated using complex dynamic models instead of just a simple formula. This is because the roll center’s position changes continuously as the vehicle moves, according to Zhang et al. [18].


$$RI=\frac{2h_{\phi }}{g}|a_{y}+gsin\phi |$$ (2)

It is essential to predict rollovers, which can provide a warning of possible rollover instability. In [19], Sellami et al. give the idea of using empirical modeling to predict the risk of rollover. The essence of this model is to use a support vector machine (SVM) algorithm. This algorithm is also applied to the heavy truck model with multiple observed variables, according to Zhu et al. [20, 21]. An algorithm to predict car rollover that uses contour lines of *RI* is introduced by Zhang et al. in [22]. The contour lines obtained from results of [22] are shown under different motion conditions, such as changing velocity. Using several solutions to estimate the sliding angle [23], or the vehicle roll angle [24] also provides a warning of rollover instability. In addition, a significant value often used when predicting rollover is the change of dynamic force, presented in [25]. Based on these predictions, warning signals are sent to the driver before a rollover accident occurs [26]. This helps the driver actively control the vehicle to avoid rollover accidents.

Modern cars have many modern anti-roll systems to limit roll instability when steering. In [27], Ding et al. suggest using an air suspension system to prevent rollover in large trucks. Other active suspension systems also help increase the vehicle’s stability under oscillation [28]. Controlling the distribution of torque to the active wheels also helps reduce vehicle rollover instability, according to Zhang et al. [29]. Active steering systems controlled by the MPC (model predictive control) algorithm provide more roll stability control than conventional steering systems [30]. In [31], Ataei et al. present braking and traction control methods for integrated stability models, including lateral and roll stability. This unique combination can help ensure stability in many directions of oscillation. For modern or autonomous vehicles, preview control methods can be applied. In this method, the surrounding signals are received by the camera, LIDAR (light detection and ranging), and sensor and then sent to the controller for further signal processing [32–34]. It is necessary to equip observers with the anti-roll control system, presented in [35] by Park et al. For studies on anti-rolling for automobiles, it is necessary to conduct experiments. This is done by Jalali et al. in [36] using a fishhook and a DLC (double land change) steering angle.

The stabilizer bar equipped with the car helps limit rolling over when steering. Most cars today are equipped with anti-roll bars. Conventional stabilizer bars are called mechanical (passive) stabilizer bars, which are made from elastomeric steel. Some advanced materials are used as an alternative to steel, such as synthetic composites, according to Masture [37]. The force generated by the passive bar can be calculated according to the displacement of the linking point [38] or the unsprung mass displacement [39]. The impact force of mechanical anti-roll bars is relatively small, so it cannot meet the roll stability requirements of the vehicle. Therefore, mechanical stabilizer bars should be replaced by active stabilizer bars controlled through modern control algorithms [40]. In [41], Dawei et al. propose designing a traditional PID (Proportional Integral Derivative) control algorithm for active anti-roll bars. A sliding-mode control rule is integrated into this controller [41]. According to Dawei et al., the roll angle and roll acceleration obtained by the sensor are the inputs of the sliding mode algorithm, while the objective of the PI algorithm is the active current and torque. A simple fuzzy algorithm is used to adjust the values of six controller parameters. However, these membership functions have only one input and are represented as normal triangular functions. The idea of designing a fuzzy algorithm for anti-roll bars is given by Sun et al. in [42]. This is an intelligent control algorithm that dynamically determines the intermediate states of the oscillation. In [43], Nguyen introduces a simple fuzzy algorithm that takes only a single input (such as roll angle, roll acceleration, etc.). The performance of the fuzzy controller, which has only one input, is poor because it ignores the influence of many other factors. An adaptive fuzzy controller with 2 inputs, including roll angle, and unsprung mass displacement is introduced in [44]. In [45], another fuzzy controller that includes two inputs applied to the active anti-roll bars is described. However, the fuzzy control rules in [44, 45] differ. In terms of [44], the membership function of the fuzzy algorithm is built on simple triangular functions with seven steps to generate 49 different fuzzy rule scenarios. For [45], the membership functions are established based on a trapezoidal function and four triangular functions with five levels corresponding to 25 fuzzy rule scenarios. Although both these algorithms are more complex than the simple fuzzy algorithm [43], their rules are generally quite simple. To improve the system’s quality, Nguyen added input to the fuzzy controller, making it a complex algorithm with three inputs [46]. The third input is the value of the unsprung mass displacement or vertical force at the wheel, which is considered in calculating the force of passive anti-roll bars [39]. The fuzzy algorithm presented in [46] suits many steering cases. Although the roll angle is reduced, the attenuation of the dynamic force at the wheel is still significant. This is improved by adjusting the fuzzy rule and the controller’s output value, as shown in [47]. With the same roll angle value, the inner wheel’s dynamic force [47] is more significant than in [46]. Although the fuzzy algorithms described in [46, 47] have three inputs, their membership function is too simple. They only have three levels, including negative, neutral, and positive. All three steps are designed based on the usual triangular function. A considerable limitation still exists in [46, 47]: the division of control states is inappropriate because of the lack of different states of the membership function. More simply, the increase in the control force of the hydraulic actuator is not controlled well (it increases too quickly or too slowly) and can affect the car’s comfort. This is a significant issue that needs to be improved. The effectiveness of active stability bars is demonstrated by experiments that describe these controllers [48, 49]. For problems involving non-parametric uncertainties and constraints, several advanced methods can be used to solve them [50, 51]. Several other control algorithms for nonlinear systems can also be applied to active anti-roll bars, such as nonlinear robust back-stepping control [52], adaptive FIT-SMC [53], the neuro-adaptive arbitrary order sliding mode [54], or PSO (particle swarm optimization) algorithms [55].

The author proposes an original fuzzy method in order to control the active stabilizer bars in this article. This algorithm takes two inputs, including the difference between the dynamic load and the body roll angle. It is appropriately inherited and selected from the algorithms outlined in [46–49]. Besides, the adaptability of the algorithm is shown through the membership function and fuzzy rule, which are entirely different from previous algorithms. This algorithm can meet the requirements of roll stability, response speed, and comfort when traveling on the road, whereas previous algorithms could only meet one of these criteria. This is an outstanding contribution to the article when compared with previous publications. The article’s content focuses on the simulation and evaluation of the results obtained from the MATLAB environment. The primary content of this article is divided into four parts. In the introduction section, some introductions and a literature review are made. In the next section (the mathematical modeling section), the design process of the dynamic model and control algorithm is shown. The process of calculating simulations and evaluating the results is described in the third section (simulation and results). Finally, some statements are made in the conclusion section.

## 2. Mathematical modeling

This section consists of two main parts: ***A vehicle model*** and ***An original fuzzy control***. The process of establishing a dynamic model is shown in the first part, while the design of the controller is illustrated in the following.

### 2.1. A vehicle model

The car dynamics model is used to describe the rolling motion of the automobile during steering. In this work, a complex model is set up to serve the simulation process. This model is a combination of many component models, including the space model, motion model, and tire model.

**Fig 1:**
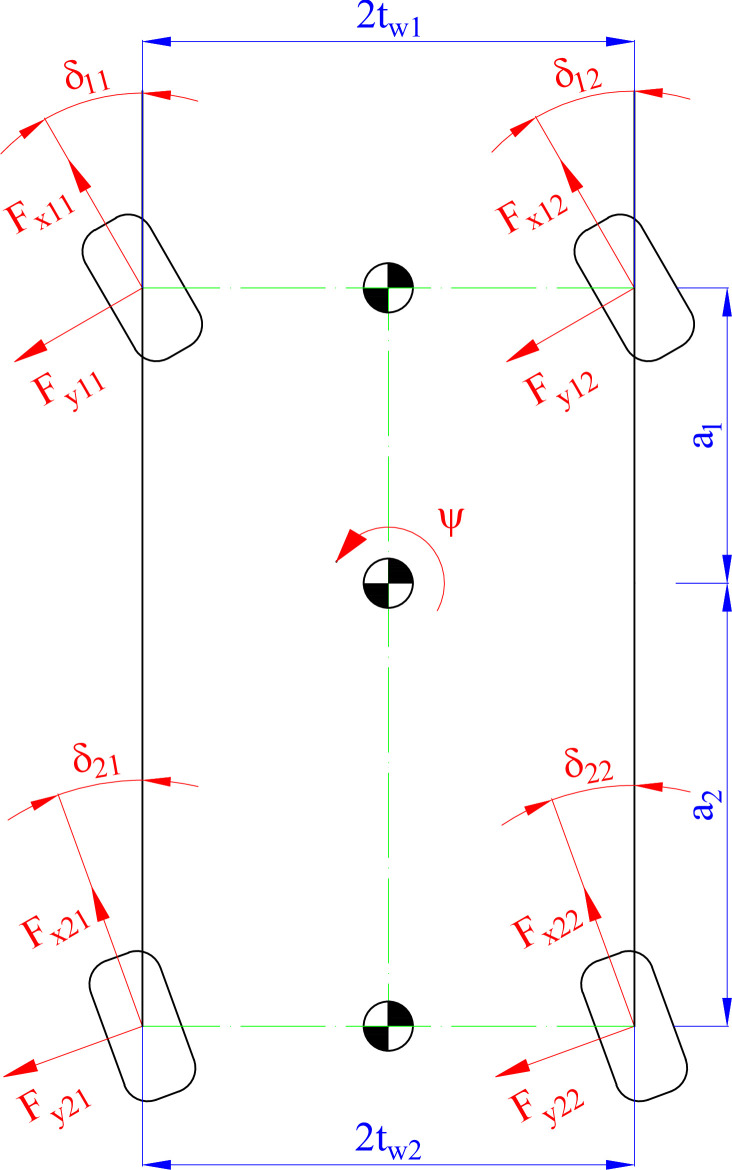
Motion model.

Consider the motion model of the car, which is shown in Fig 1. Eqs (3) and (4) describe the longitudinal and lateral motions of the car, while the yaw motion is illustrated by Eq (5).


$$F_{ix}=\sum_{i,j=1}^{2}\left(F_{xij}cos\delta_{ij}-F_{yij}sin\delta_{ij}\right)+F_{cex}$$ (3)

$$F_{iy}=\sum_{i,j=1}^{2}\left(F_{xij}sin\delta_{ij}+F_{yij}cos\delta_{ij}\right)-F_{cey}$$ (4)

$$M_{\psi }=\sum_{i,j=1}^{2}\left[{\left(-1\right)}^{j}(F_{xij}cos\delta_{ij}-F_{yij}sin\delta_{ij})t_{wi}+{\left(-1\right)}^{i+1}(F_{xij}sin\delta_{ij}+F_{yij}cos\delta_{ij})a_{i}-M_{zij}\right]$$ (5)

The linking Eqs from (6) to (10) help determine the unknowns in Eqs from (3) to (5). Some more detailed explanations should be consulted in [56, 57].


$$F_{ix}=(m_{s}+\sum_{i,j=1}^{2}m_{uij}){\dot{v}}_{x}$$ (6)

$$F_{iy}=(m_{s}+\sum_{i,j=1}^{2}m_{uij}){\dot{v}}_{y}$$ (7)

$$F_{cex}=(m_{s}+\sum_{i,j=1}^{2}m_{uij})(\dot{\beta }+\dot{\psi })v_{y}$$ (8)

$$F_{cey}=(m_{s}+\sum_{i,j=1}^{2}m_{uij})(\dot{\beta }+\dot{\psi })v_{x}$$ (9)

$$M_{\psi }=J_{\psi }\ddot{\psi }$$ (10)

The relationship between longitudinal and lateral velocity is shown through the heading angle *β*, according to the Eq (11).


$$\beta =arctan\frac{v_{y}}{v_{x}}$$ (11)

The moments and forces of wheels are calculated based on tire models. In this study, the author uses the Pacejka tire model to determine the values of *F*_*x*_, *F*_*y*_, and *M*_*z*_ [56]. The symbols for Eqs (12), (13), and (14) should be referred to in [56].


$$F_{x}=D_{x}sin[C_{x}artan(B_{x}\kappa_{x})]+S_{vx}$$ (12)

$$F_{y}=D_{y}sin[C_{y}artan(B_{y}\kappa_{y})]+S_{vy}$$ (13)

$$M_{z}=D_{z}sin[C_{z}artan(B_{z}\kappa_{z})]+S_{vz}$$ (14)

Eqs (3) to (14) are established to help determine the value of the lateral acceleration *a*_*y*_ produced when the vehicle is steering.


$$a_{y}={\dot{v}}_{y}+(\dot{\psi }+\dot{\beta })v_{x}$$ (15)

**Fig 2:**
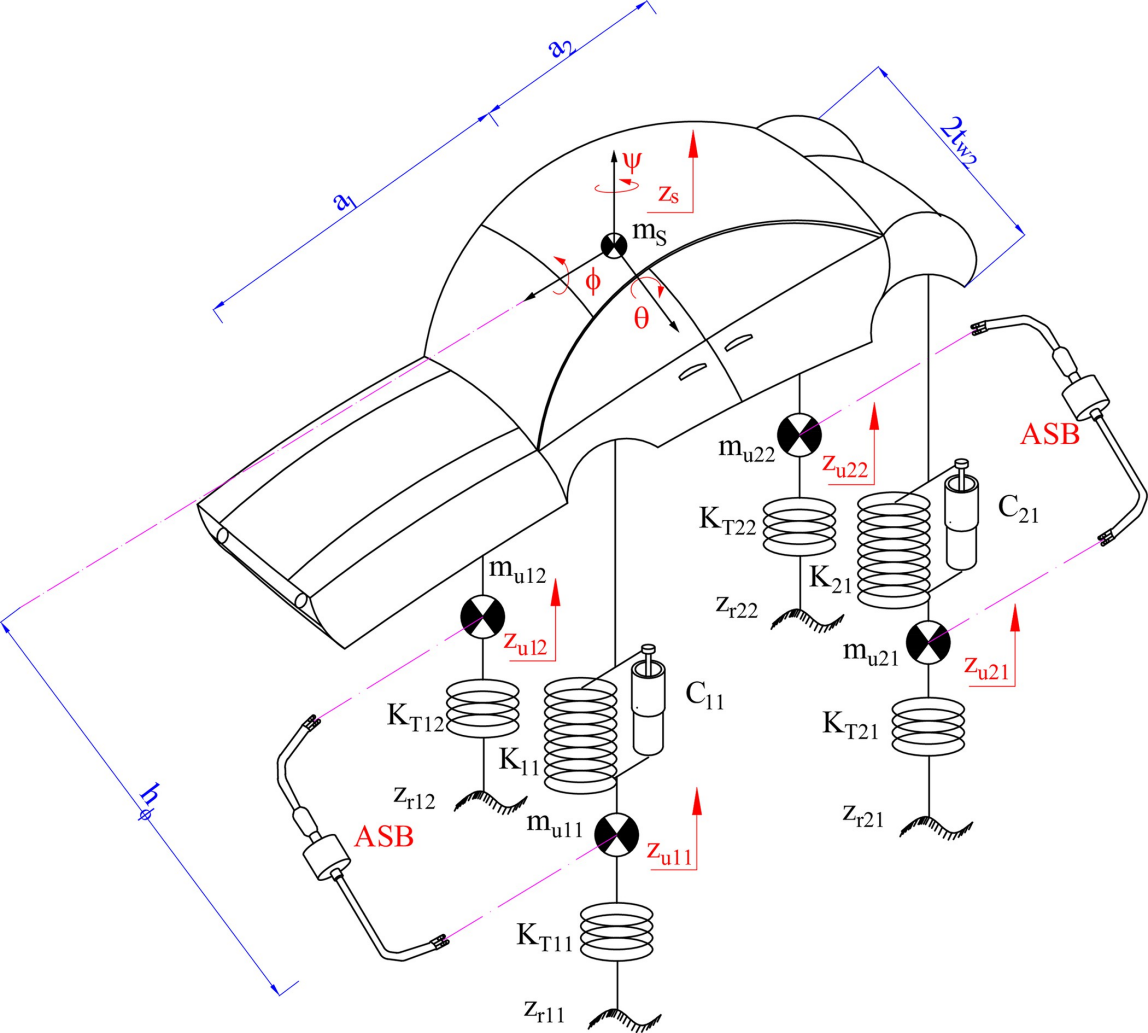
Spatial model.

The vehicle rollover oscillations are described through a spatial dynamics model with 7 DOFs (Fig 2). Applying D’Alembert’s principle, Eqs from (16) to (19) describe the car’s rollover oscillation (see in [56]).


$$F_{ims}=\sum_{i,j=1}^{2}\left(F_{Kij}+F_{Cij}\right)$$ (16)

$$M_{\phi }=\sum_{i,j=1}^{2}\left[{\left(-1\right)}^{j-1}(F_{Kij}+F_{Cij})t_{wi}\right]+\left\{gsin\phi +a_{y}cos\phi \right\}m_{s}h_{\phi }$$ (17)

$$M_{\theta }=\sum_{i,j=1}^{2}{\left(-1\right)}^{i-1}(F_{Kij}+F_{Cij})a_{i}$$ (18)

$$F_{imuij}=F_{KTij}-F_{Kij}-F_{Cij}+{\left(-1\right)}^{j}F_{ASBij}$$ (19)

Where:

$$F_{ims}=m_{s}{\ddot{z}}_{s}$$ (20)

$$F_{imuij}=m_{uij}{\ddot{z}}_{uij}$$ (21)

$$F_{Kij}=K_{ij}[z_{s}-z_{uij}+{\left(-1\right)}^{j+1}t_{wi}\phi +{\left(-1\right)}^{i+1}a_{i}\theta ]$$ (22)

$$F_{Cij}=C_{ij}[{\dot{z}}_{s}-{\dot{z}}_{uij}+{\left(-1\right)}^{j+1}t_{wi}\dot{\phi }+{\left(-1\right)}^{i+1}a_{i}\dot{\theta }]$$ (23)

$$F_{KTij}=K_{Tij}(z_{rij}-z_{uij})$$ (24)

$$M_{\phi }=(J_{\phi }+m_{s}h_{\phi }^{2})\ddot{\phi }$$ (25)

$$M_{\theta }=(J_{\theta }+m_{s}h_{\theta }^{2})\ddot{\theta }$$ (26)

**Fig 3:**
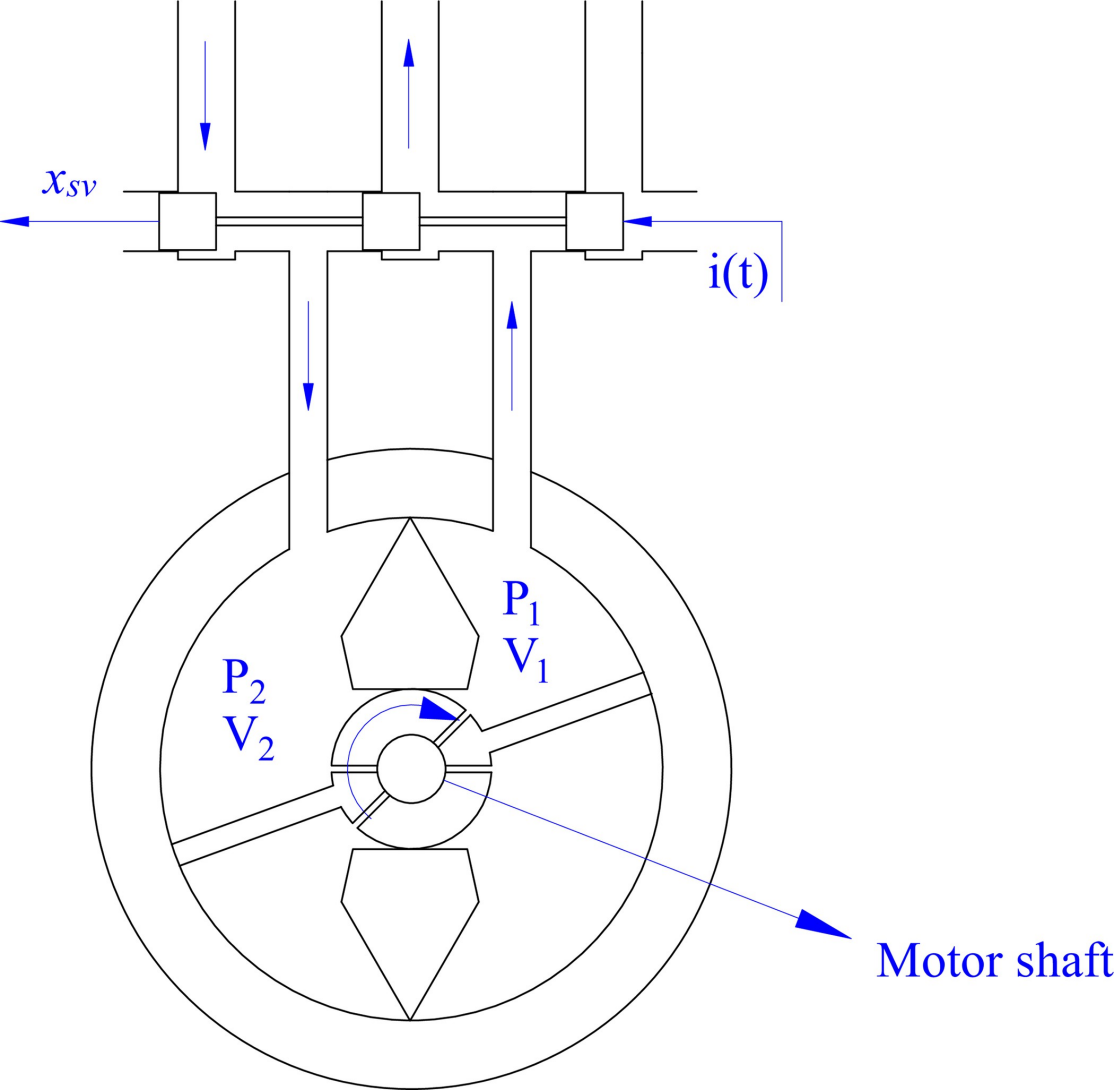
Hydraulic actuator.

The impact force of active stabilizer bars (*F*_*ASB*_) is produced by the fluid pressure difference inside a hydraulic actuator (Fig 3).


$$F_{ASB}=\frac{M_{ASB}}{r}$$ (27)

$$M_{ASB}=D_{m}\Delta P$$ (28)

The operating process of a hydraulic actuator is described through Eqs (29) and (30).


$$D_{m}\Delta P=J_{m}{\ddot{\phi }}_{m}+B_{m}{\dot{\phi }}_{m}+T_{r}$$ (29)

$$k_{qi}x_{sv}=D_{m}{\dot{\phi }}_{m}+k_{ce}\Delta P+\frac{V}{4\beta_{e}}\Delta \dot{P}$$ (30)

The servo valve displacement is dependent on the current supplied. The regulator plays an essential role in calculating the control current for the system. The relationship between the control signal and the valve displacement is described as follows:

$${\dot{x}}_{sv}\tau =k_{sv}i(t)-x_{sv}$$ (31)

The control signal is defined by a controller, designed in the next part.

### 2.2. An original fuzzy control

The fuzzy algorithm plays an essential role in controlling the behavior of the stabilizer bars. Different from other common control algorithms, this intelligent control algorithm is model-independent; however, the operation of the anti-roll bar, which is controlled by the fuzzy algorithm, is dependent on vehicle oscillation. The use of dynamic models helps evaluate and demonstrate the operability of the stabilizer bar more clearly and precisely. For the most part, fuzzy algorithms are designed based on the experience of researchers. Besides, several criteria are also given when designing the controller to help enhance the system’s quality.

**Fig 4:**
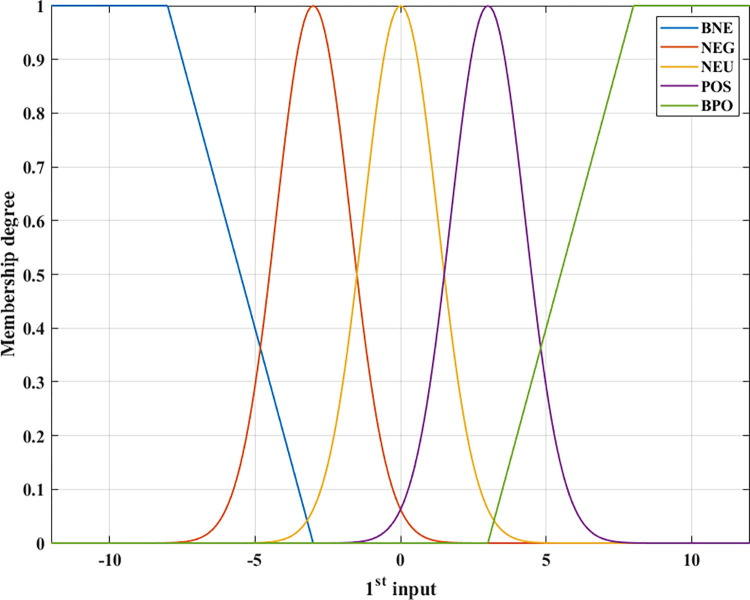
Membership function (1^st^ input).

**Fig 5:**
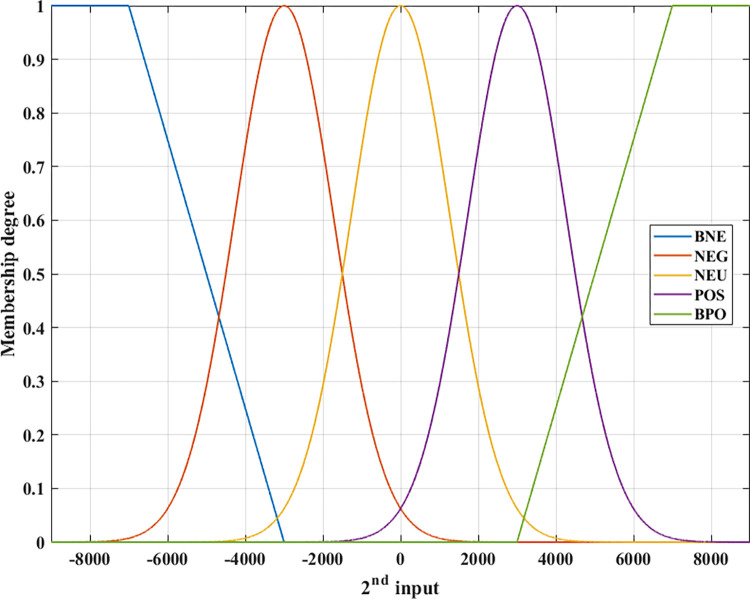
Membership function (2^nd^ input).

In this article, the author proposes to design the original fuzzy algorithm with two inputs for an automotive stabilizer bar control system. Two inputs to the controller include signals related to the roll angle and the change in the load on wheels during steering. The output of the membership function is the membership degree, which ranges from 0 ÷ 1. Combined with the fuzzy rule, the final output of the controller is the current signal. The membership function is depicted in Figs 4 and 5 with five levels: BNE (big negative); NEG (negative); NEU (neutral); POS (positive); and BPO (big positive), and it is more than [46, 47]. There are two types of membership functions: TMF (trapezoidal membership function) and GMF (Gaussian membership function). These functions are described through mathematical Eqs (32) and (33) for TMF and GMF, respectively. Using the GMF function helps to change the control signal more stably, avoiding overshoots if only triangular functions are used [41, 44–47].


$$\mu_{MF}(x)=\{\begin{array}{ll} 0 & x\leq a\\ \frac{x-a}{b-a} & a<x<b\\ 1 & b<x<c\\ 0 & x\geq d \end{array}$$ (32)

$$\mu_{MF}(x)=e^{-\frac{1}{2}{\left(\frac{x-e}{\sigma }\right)}^{2}}$$ (33)

Where: *a*, *b*, *c*, *d*, *e*, *σ* are the respective values of functions.

The membership functions are defined according to points:

○ If the car’s roll angle is large, the control signal must be significant, and vice versa.
○ If the change in the wheel dynamic load is large, the control signal must be significant, and vice versa.
○ In the safe domain, avoiding a sudden increase in the control signal is necessary.
○ In the dangerous domain, the control signal change must be fast and accurate.
○ The magnitude of the control signal should be limited.

According to these views, two membership functions corresponding to two input signals are shown in Figs 4 and 5. The TSK (Takagi-Sugeno-Kang) fuzzy model is used for the object. The defuzzification process is determined using the WTAVER (weighted average) formula.


$$y'=\frac{\sum_{i=1}^{n}\alpha_{i}y_{i}}{\sum_{i=1}^{n}\alpha_{i}}$$ (34)

**Fig 6:**
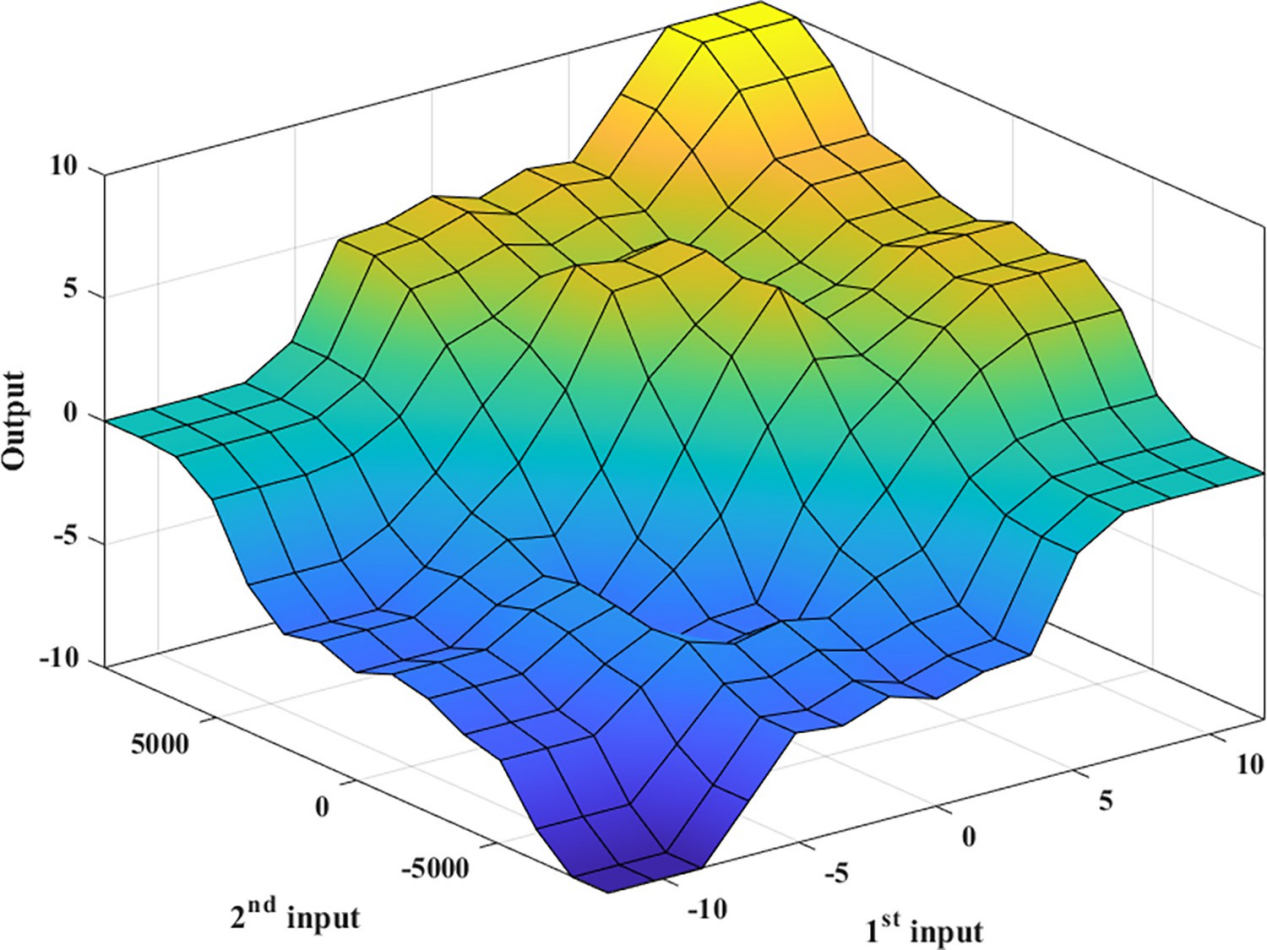
Fuzzy surface.

Fuzzy rules are shown in Table 1. A clearer illustration of fuzzy rules is shown in Fig 6, which shows the relationship between two input signals and a single output signal. The point of view of fuzzy rule selection is similar to that of the design of membership functions. These views also meet the speed of response, smoothness, and roll stability criteria. Besides, the determination of fuzzy rules is based on the previous calculation and simulation process, which has been repeated many times to choose the appropriate rules.

**Table 1:** Fuzzy rules.

No	1^st^ input	2^nd^ input	Output	No	1^st^ input	2^nd^ input	Output
1	BNE	BNE	BNE	14	NEU	POS	POS
2	BNE	NEG	NEG	15	NEU	BPO	POS
3	BNE	NEU	NEG	16	POS	BNE	NEG
4	BNE	POS	NEG	17	POS	NEG	NEU
5	BNE	BPO	NEU	18	POS	NEU	POS
6	NEG	BNE	NEG	19	POS	POS	POS
7	NEG	NEG	NEG	20	POS	BPO	POS
8	NEG	NEU	NEG	21	BPO	BNE	NEU
9	NEG	POS	NEU	22	BPO	NEG	POS
10	NEG	BPO	POS	23	BPO	NEU	POS
11	NEU	BNE	NEG	24	BPO	POS	POS
12	NEU	NEG	NEG	25	BPO	BPO	BPO
13	NEU	NEU	NEU				

The simulation, calculation, and evaluation process will be continued in the next section.

## 3. Simulation and results

### 3.1. Simulation condition

**Fig 7:**
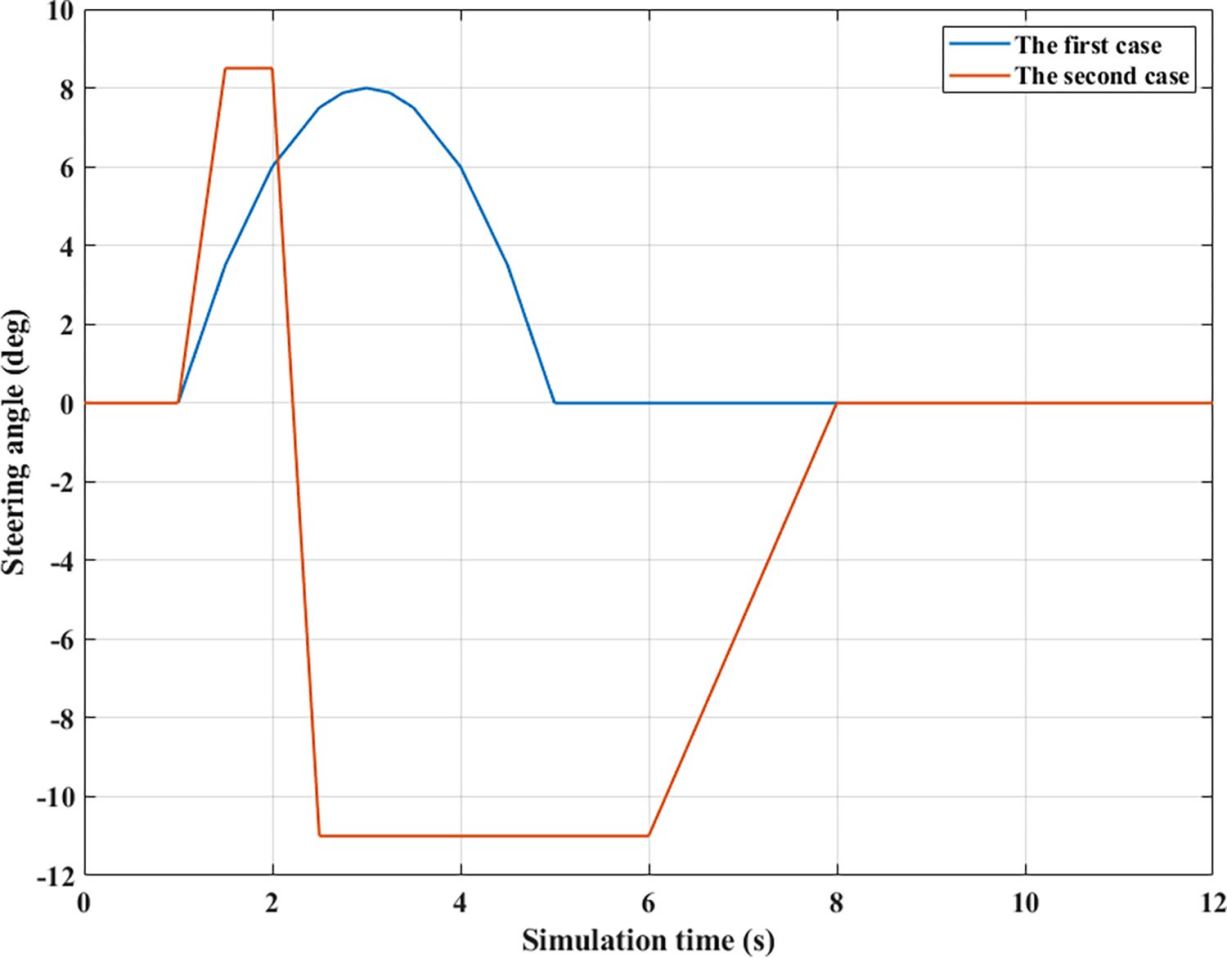
Steering angle.

Simulation conditions need to be determined before conducting simulations and calculations. Steering angle and movement speed are the input values of the simulation process. Two steering angles are used in this study for the first and second cases, respectively (Fig 7). The speed of the car is investigated through three values, *v*_*1*_ = 70 (km/h), *v*_*2*_ = 80 (km/h), and *v*_*3*_ = 90 (km/h). The simulation results are compared between 3 situations: cars using active stabilizer bars (Fuzzy Active); cars using passive stabilizer bars (Passive); and cars without stabilizer bars (None).

The parameters of the vehicle that serve the simulation are introduced in Table 2.

**Table 2:** Vehicle specifications.

No	Description	Value	Unit	Symbol
1	Sprung mass	1750	kg	*m*_*s*_
2	Unsprung mass	48	kg	*m*_*u*_
3	Half of track width	730	mm	*t*_*w*_
		725		
4	Longitudinal distance from CG	1240	mm	*a*
		1680		
5	Roll axis distance	560	mm	*h*_*ϕ*_
6	Roll inertia moment	690	kgm^2^	*J*_*ϕ*_
7	Pitch inertia moment	2580	kgm^2^	*J*_*θ*_
8	Yaw inertia moment	2670	kgm^2^	*J*_*ψ*_
9	Spring coefficient	35550	N/m	*K*
		34000		
10	Damper coefficient	3130	Ns/m	*C*
		3100		
11	Tire coefficient	173000	N/m	*K*_*T*_
12	Gravitational acceleration	9.81	m/s^2^	*g*

### 3.2. Results and discussion

#### 3.2.1. The first case

*v*_*1*_ *= 70 (km/h)*. According to Fig 7, the steering angle in the first case implies that the car tends to change directions of motion. When the steering angle is zero, the car goes straight, and output values are all zero. Since the steering angle value rises, the output values also change accordingly.

**Fig 8:**
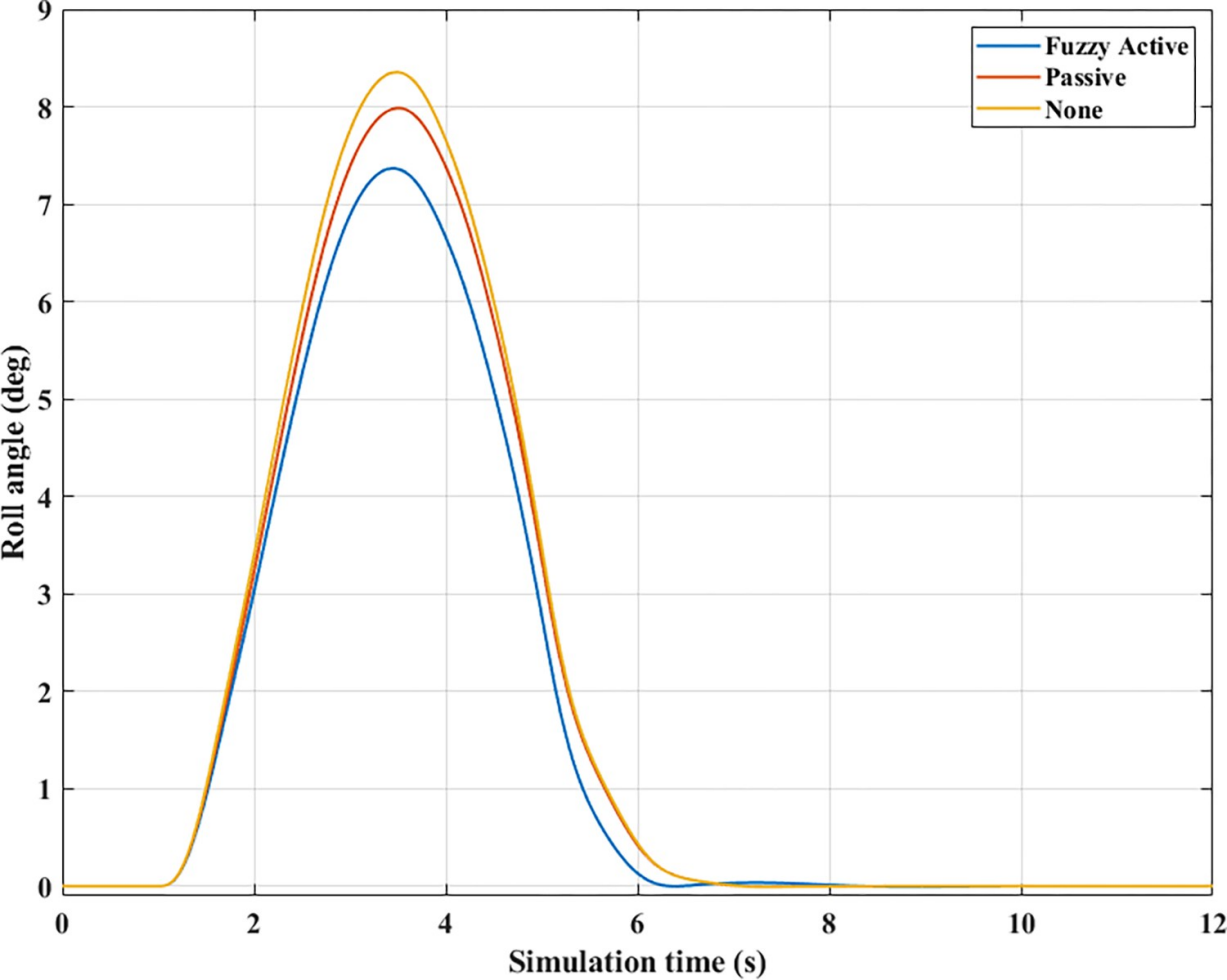
Roll angle (1^st^ case–v_1_).

**Fig 9:**
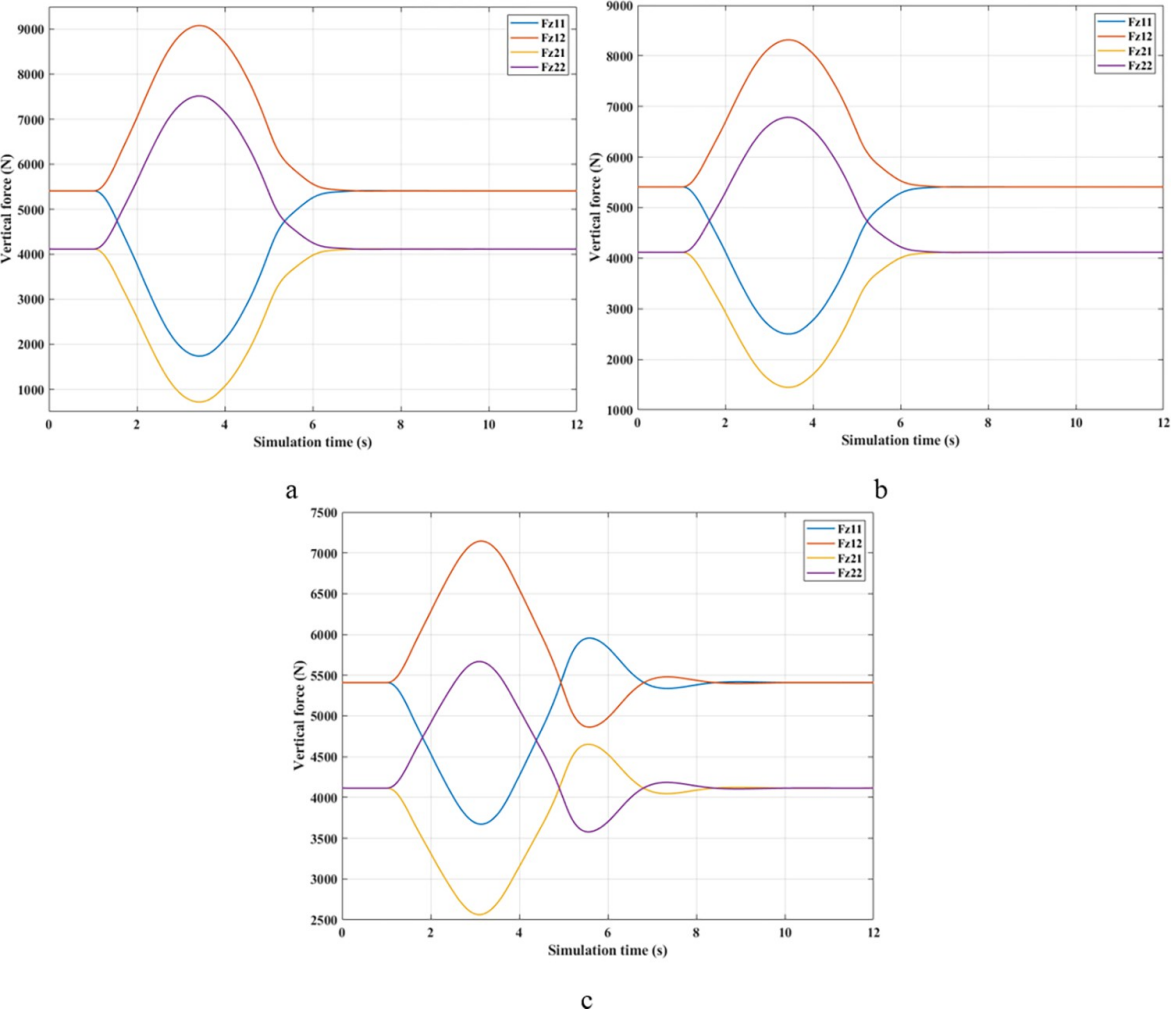
Vertical force (1^st^ case–v_1_). a) None; b) Passive; c) Fuzzy Active.

The change of roll angle is an important object that needs attention when studying the dynamic behavior of automobiles. According to the findings illustrated in Fig 8, the roll angle values increase gradually from the first second until they peak (Fuzzy Active: 7.37°, Passive: 7.98°, and None: 8.35°). These values are obtained when the vehicle travels at a steady speed, *v*_*1*_ = 70 (km/h). The increased roll angle is responsible for the difference in dynamic forces between the wheel’s two sides, as depicted in Fig 9.

**Fig 10:**
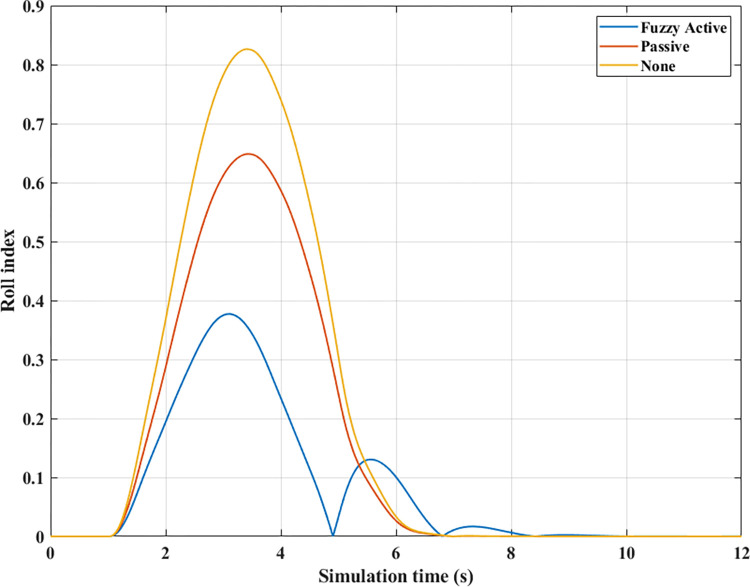
Roll index (1^st^ case–v_1_).

Fig 9A depicts the change in the vertical forces of wheels when changing the direction of motion at a speed of *v*_*1*_ = 70 (km/h) without the anti-roll bar. Based on this result, the minimum value of the vertical force belongs to the wheel position (21), i.e., the wheel is on the rear left-hand side. This is entirely consistent with reality. Its minimum is 715.28 (N), a low value corresponding to a rollover index *RI* = 0.83 (Fig 10). The variation of the dynamic force at wheels when the car uses a passive stabilizer is shown in Fig 9B. Comparing results between Fig 9A and 9B, it is easy to see that the minimum force value is improved when the car uses the stabilizer bars, F_z21min_ = 1447.33 (N). Reducing the load difference between wheels help bring the value of *RI* to a stable threshold (*RI* = 0.65), which reduces the risk of rollover (Fig 10).

Compared with above 2 situations, the value of the dynamic force in the 3^rd^ situation (Fig 9C) is much smaller. Once the automobile uses active stability bars controlled by a novel fuzzy algorithm, the value of the dynamic force is raised to 2563.85 (N). As a result, the interaction between the road and wheels is more ensured, leading to a drastic reduction in the risk of rollovers (RI = 0.38).

**Fig 11:**
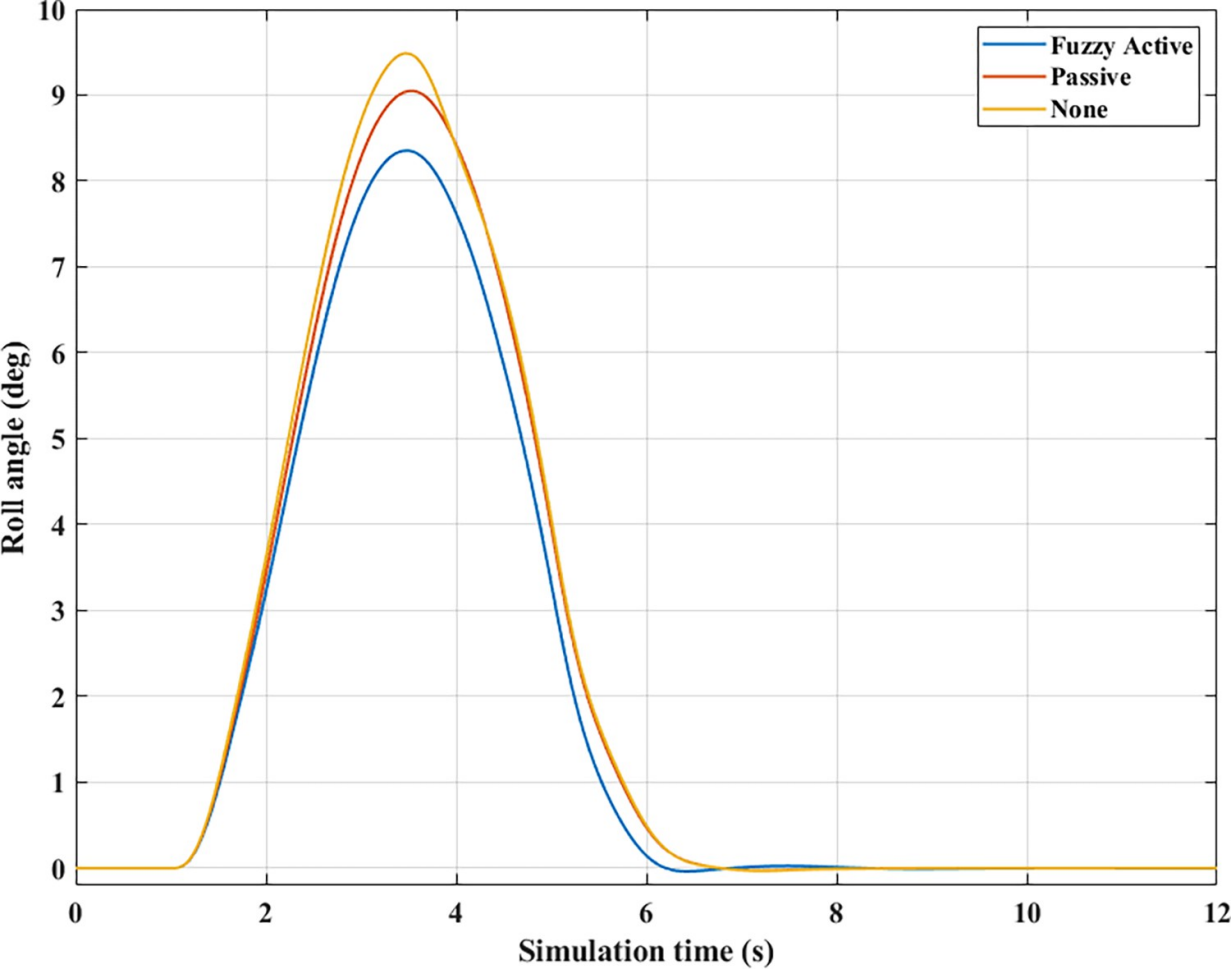
Roll angle (1^st^ case–v_2_).

*v*_*2*_ *= 80 (km/h)*. As the automobile’s velocity rises to a value of *v*_*2*_ = 80 (km/h), a reasonable prediction shows that both the roll index and the roll angle increase while the minimum force value drops sharply. The trend changes in the roll angle in Fig 11 are similar to those in Fig 8; however, their values are more significant. According to simulation findings, the peak value of roll angle when an automobile is traveling at speed *v*_*2*_ is 9.49°, 9.05°, and 8.35°, respectively, corresponding to three situations: (None), (Passive), and (Fuzzy Active). The phase of the (Passive) situation is slightly behind the other two because the response speed of the passive stabilizer is slow. In contrast, the response of the active stabilizer is more.

**Fig 12:**
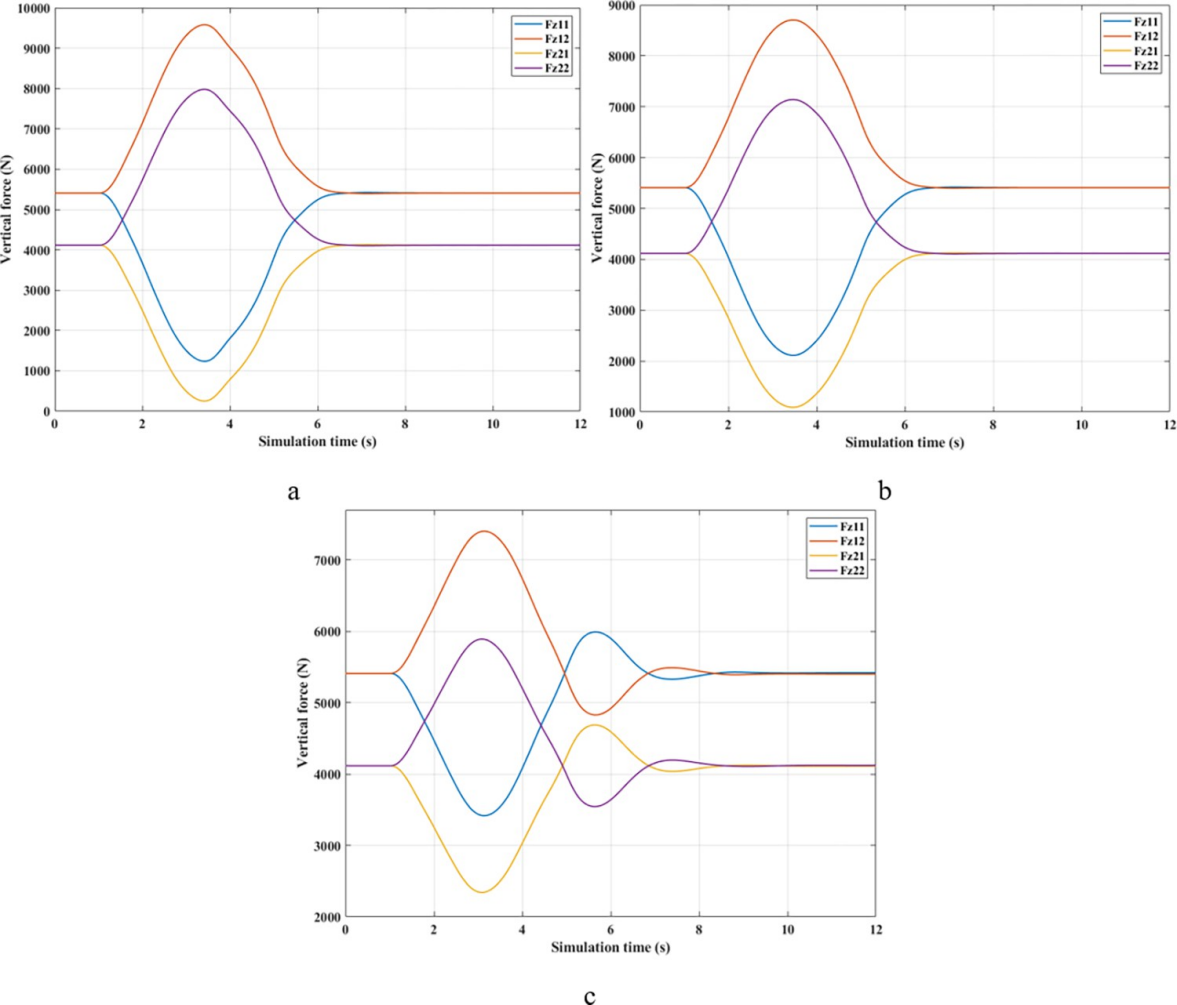
Vertical force (1^st^ case–v_2_). a) None; b) Passive; c) Fuzzy Active.

**Fig 13:**
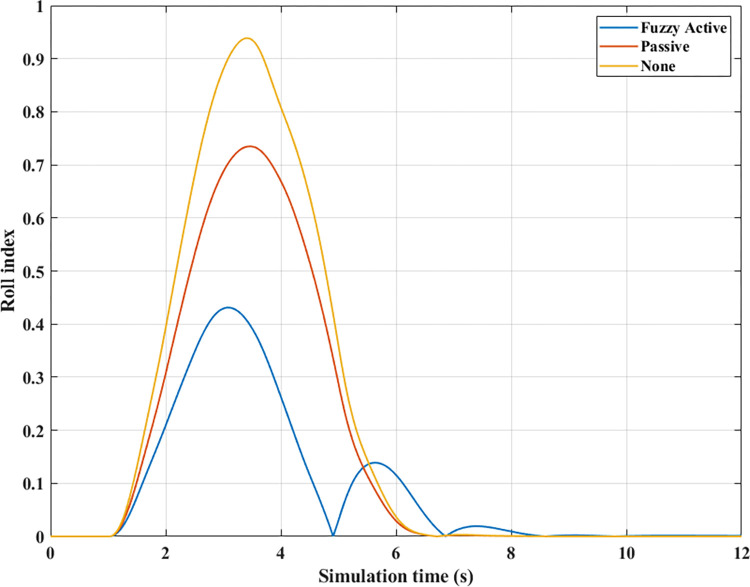
Roll index (1^st^ case–v_2_).

As predicted above, the change in wheel dynamics when the car is traveling at speed *v*_*2*_ is more significant than that at speed *v*_*1*_ (Fig 12). According to Fig 12A, the dynamic force at the left rear wheel is strongly declined to only 252.43 (N). This shallow value warns of a possible 94% rollover risk based on *RI* criteria (Fig 13). This is further improved by using mechanical stabilizer bars, which raise the minimum force value to 1091.28 (N) (Fig 12B). Increasing the vertical force value reduces the risk of rollover, i.e., reduces the value of *RI* (only 0.73).

Although passive anti-roll bars can partially limit rollover, their effectiveness is not high. If an automobile is equipped with active stability bars, the roll stabilization effect can be more ensured by applying the fuzzy control algorithm to this anti-roll system. According to Fig 12C, the minimum force value at the wheel position (21) is only 2133.11 (N), corresponding to a value of *RI* of 0.48. With the slight change in this situation, the vehicle’s roll stability is always well maintained.

In the third situation (Fuzzy Active), the car oscillation goes through more phases than in the previous two. The primary oscillation is made in the first phase, while the later phases are only minor. The cause of this phenomenon is the influence of the inertia of the active stabilizer bar, which exerts a large force to act on the unsprung mass. This causes the dynamic force to change for a short amount of time after the steering is over. Ultimately, these values still converge to be similar to the first and second situations, just that there is still a certain delay.

**Fig 14:**
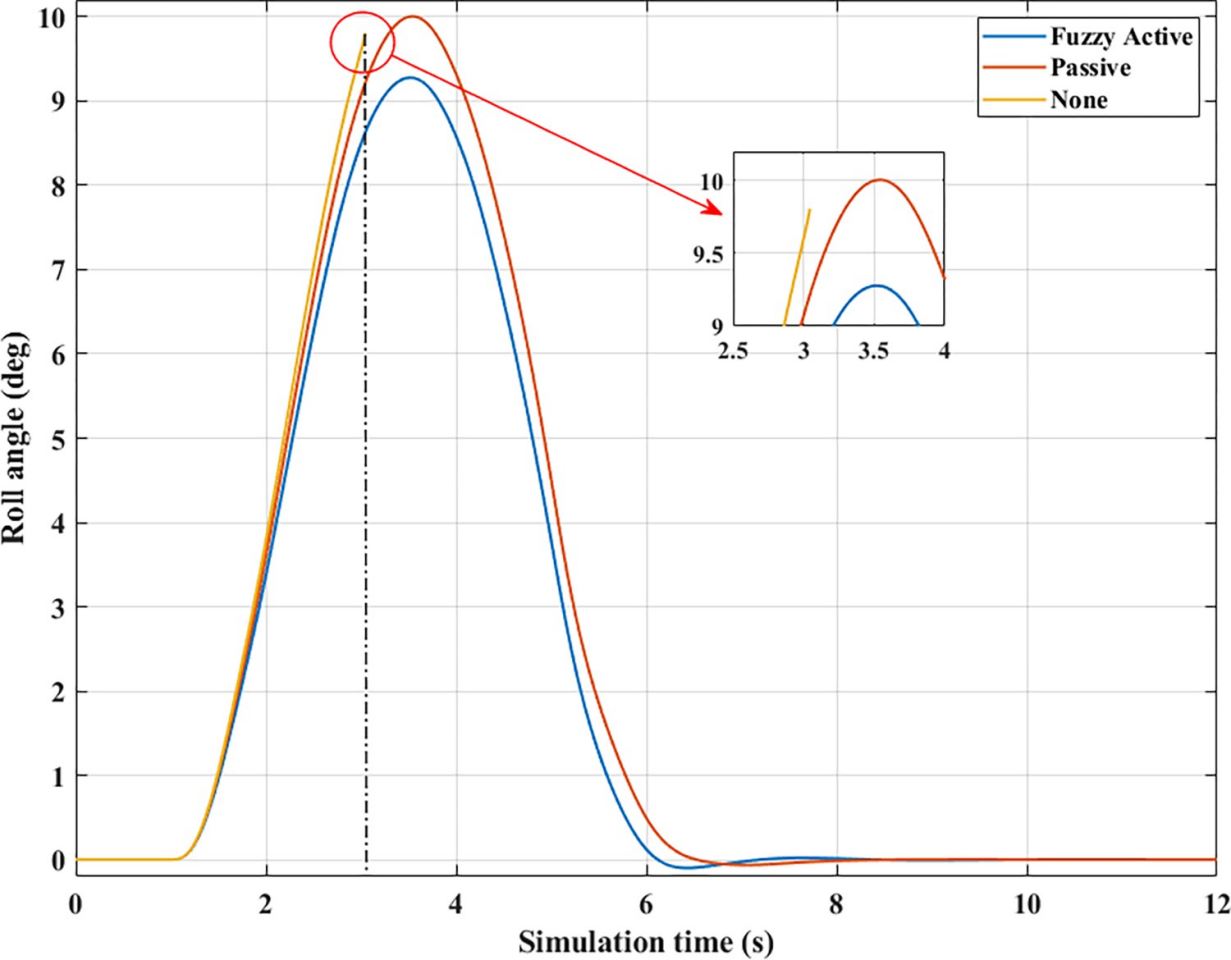
Roll angle (1^st^ case–v_3_).

*v*_*3*_ *= 90 (km/h)*. Roll instability can happen if the car’s speed increases while the steering angle stays the same. According to Fig 14, the peak value of the roll angle when an automobile does not have the anti-roll bar is only 9.81°, which is achieved before the rollover occurs, t = 3.05 (s). However, if the automobile is equipped with passive stabilizer bars on both axles, rollover phenomenon does not occur when moving at speed *v*_*3*_, although its maximum roll angle is quite large, up to 10.01°. The value of the (Fuzzy Active) situation is lower, only 9.28°.

**Fig 15:**
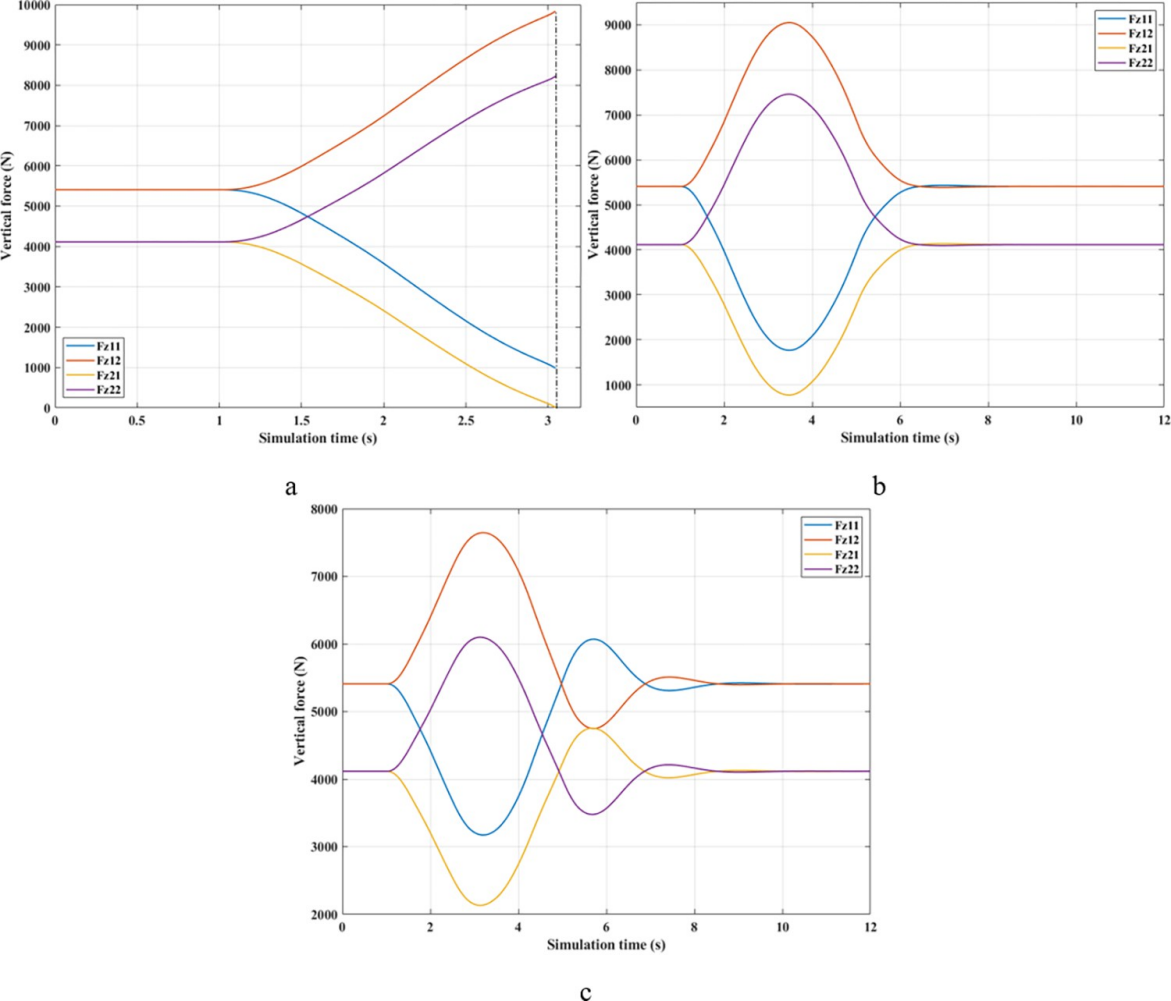
Vertical force (1^st^ case–v_3_). a) None; b) Passive; c) Fuzzy Active.

**Fig 16:**
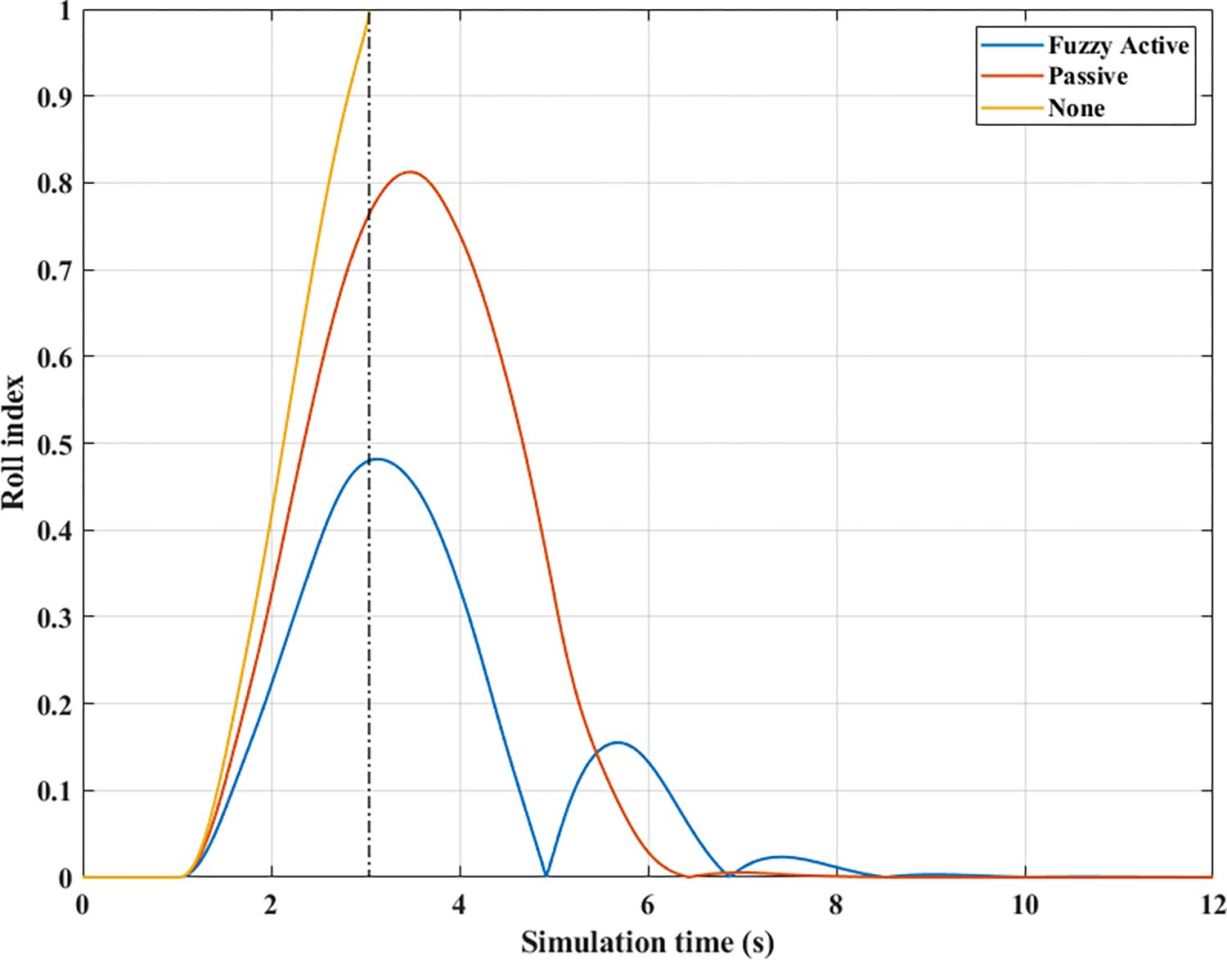
Roll index (1^st^ case–v_3_).

The change of dynamic force occurs more strongly when the vehicle moves at high speed, *v*_*3*_ = 90 (km/h). According to Fig 15A, the value of the dynamic force approaches zero when rollover occurs in a situation where the automobile does not have any anti-roll bars. Meanwhile, this value is only 773.06 (N) for the (Passive) situation (Fig 15B), corresponding to a rolling index (*RI*) of 0.81 (Fig 16). Usually, if the roll index is more significant than 0.8, it is considered a danger warning. The active stabilizer bar helps limit rollover hazards when it is controlled by an original fuzzy algorithm. According to Fig 15C, the value of the dynamic force remains at a constant level, F_z21min_ = 2133.11 (N), about 2.76 times larger than the (Passive) situation. Roll stability is maintained at a safety margin, which is demonstrated by the value of the roll index, *RI* = 0.48.

#### 3.2.2. The second case

The first case only uses simple steering with small amplitude, so the rollover occurs only in the (None) situation at *v*_*3*_. In the second case, the steering angle is the fish-hook type with larger amplitude, but the survey speed still passes through three thresholds: *v*_*1*_, *v*_*2*_, and *v*_*3*_. The type of fish-hook steering angle has two phases; the steering angles of the two phases can be the same or different. Usually, the fish-hook steering angle has a more significant and dangerous second phase than the first phase.

**Fig 17:**
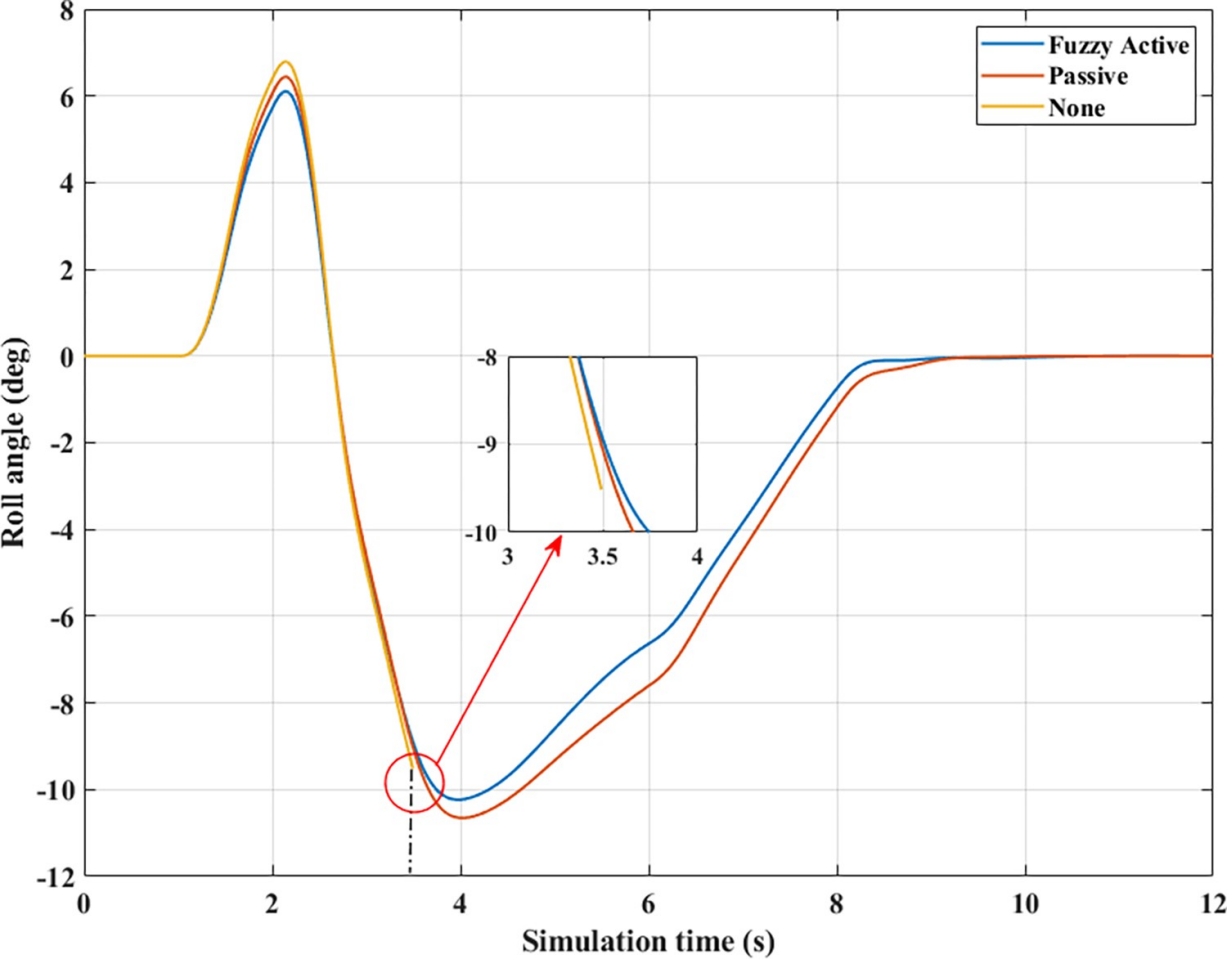
Roll angle (2^st^ case–v_1_).

*v*_*1*_ *= 70 (km/h)*. At average speed, *v*_*1*_ = 70 (km/h), the change in the roll angle depicted in Fig 17 corresponds to three test scenarios: (None), (Passive), and (Fuzzy Active). Even though the car only travels at a low speed, rollover still occurs when the automobile does not have an anti-roll bar. At time t = 3.49 (s), the maximum roll angle reaches 9.52° before the car rollover occurs. This phenomenon is more prevented if passive and active stabilizer bars are used. The peak value of roll angle of the car can be up to 10.65° and 10.23° for (Passive) and (Fuzzy Active), respectively, which is larger than the first situation and still does not roll over.

**Fig 18:**
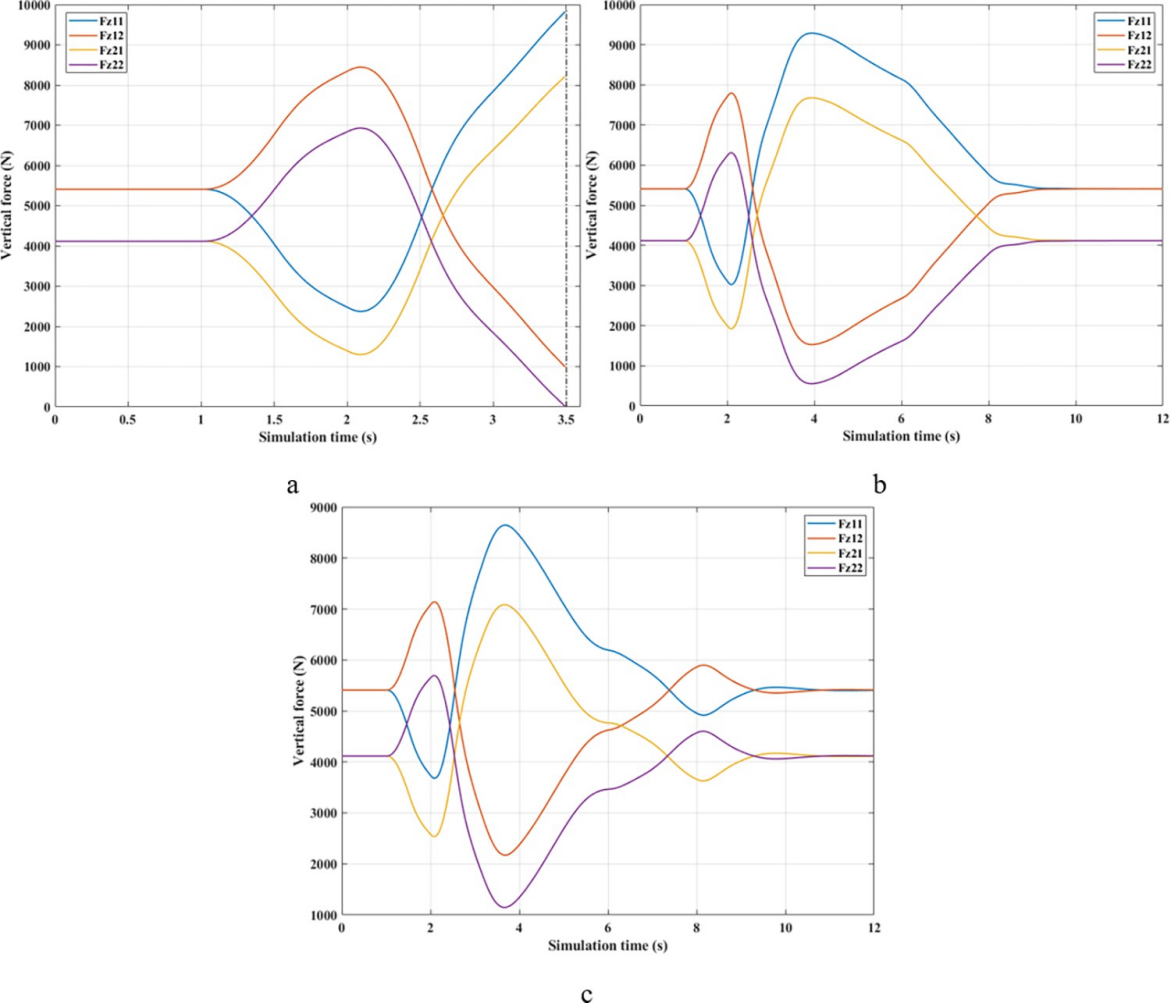
Vertical force (2^st^ case–v_1_). a) None; b) Passive; c) Fuzzy Active.

**Fig 19:**
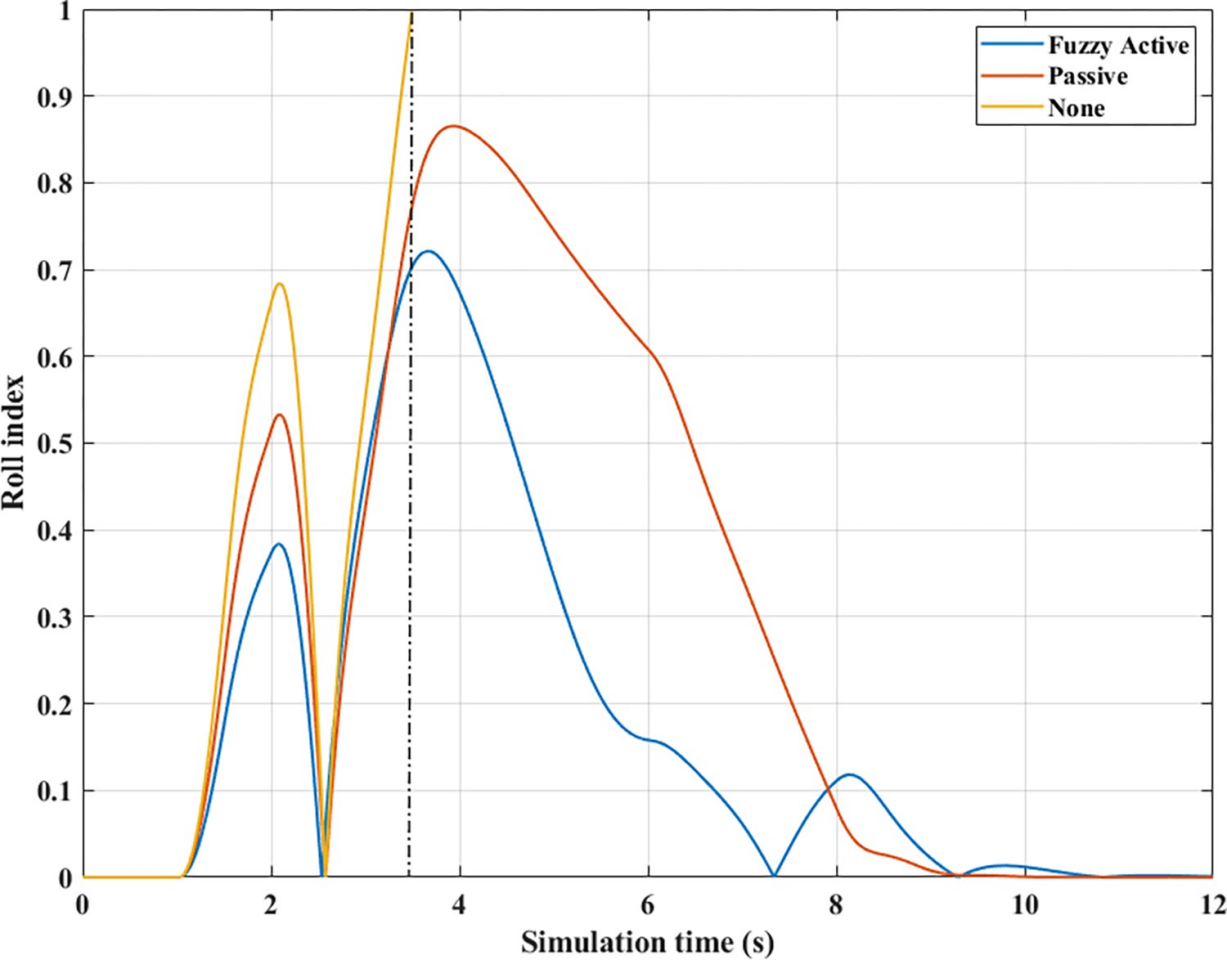
Roll index (2^st^ case–v_1_).

The phenomenon of car rollover is more clearly described by the change in dynamic forces at wheels. The dynamic force value at the wheel approaches zero when the automobile does not use a stability bar. However, the minimum value of the dynamic force is only 554.61 (N) when passive anti-roll bars are used. Rollover does not occur in the second situation (Passive), but this is also a dangerous threshold, as indicated by the risk of rolling over as high as 87% (Fig 19). The car’s stability is always well guaranteed when using hydraulic stability bars directed by the novel fuzzy solution designed in this research. According to results in Fig 18C, the minimum force value at the wheel (21) is only 1147.25 (N). This ensures good interaction between the road and wheels, and no roll instability occurs because the *RI* is only 0.71 (Fig 19).

**Fig 20:**
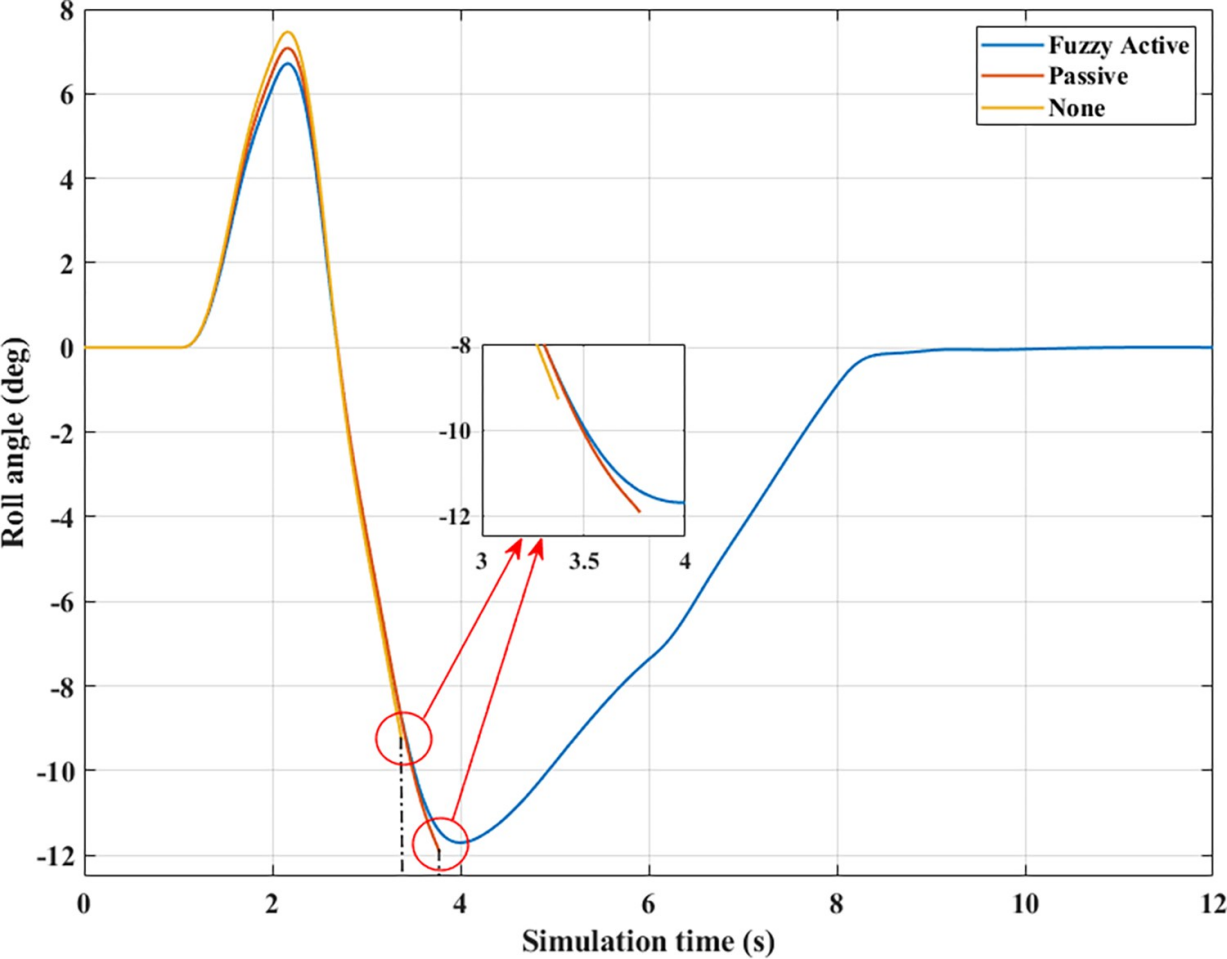
Roll angle (2^st^ case–v_2_).

*v*_*2*_ *= 80 (km/h)*. Increased speeds can lead to many rollover stability problems, even when mechanical stabilizer bars are fitted. The change of roll angle value during the two phases of oscillation is depicted in Fig 20. According to this finding, the maximum roll angles obtained in the first phase of the oscillation are 7.47°, 7.08°, and 6.72°, corresponding to three situations (Fuzzy Active), (Passive), and (None). Overall, this difference is not too big. The effect of active stability bars directed by a new fuzzy solution is more clearly demonstrated in the 2^nd^ phase of the oscillation. The study’s findings showed that rollover occurs in (None) and (Passive), while the other situation remains stable. The peak values of the roll angle in the second phase are 9.29° and 11.93° for two rollover situations, in the same order as above. Meanwhile, the car’s roll angle may be up to 11.70° without a rollover accident when using active anti-roll bars.

**Fig 21:**
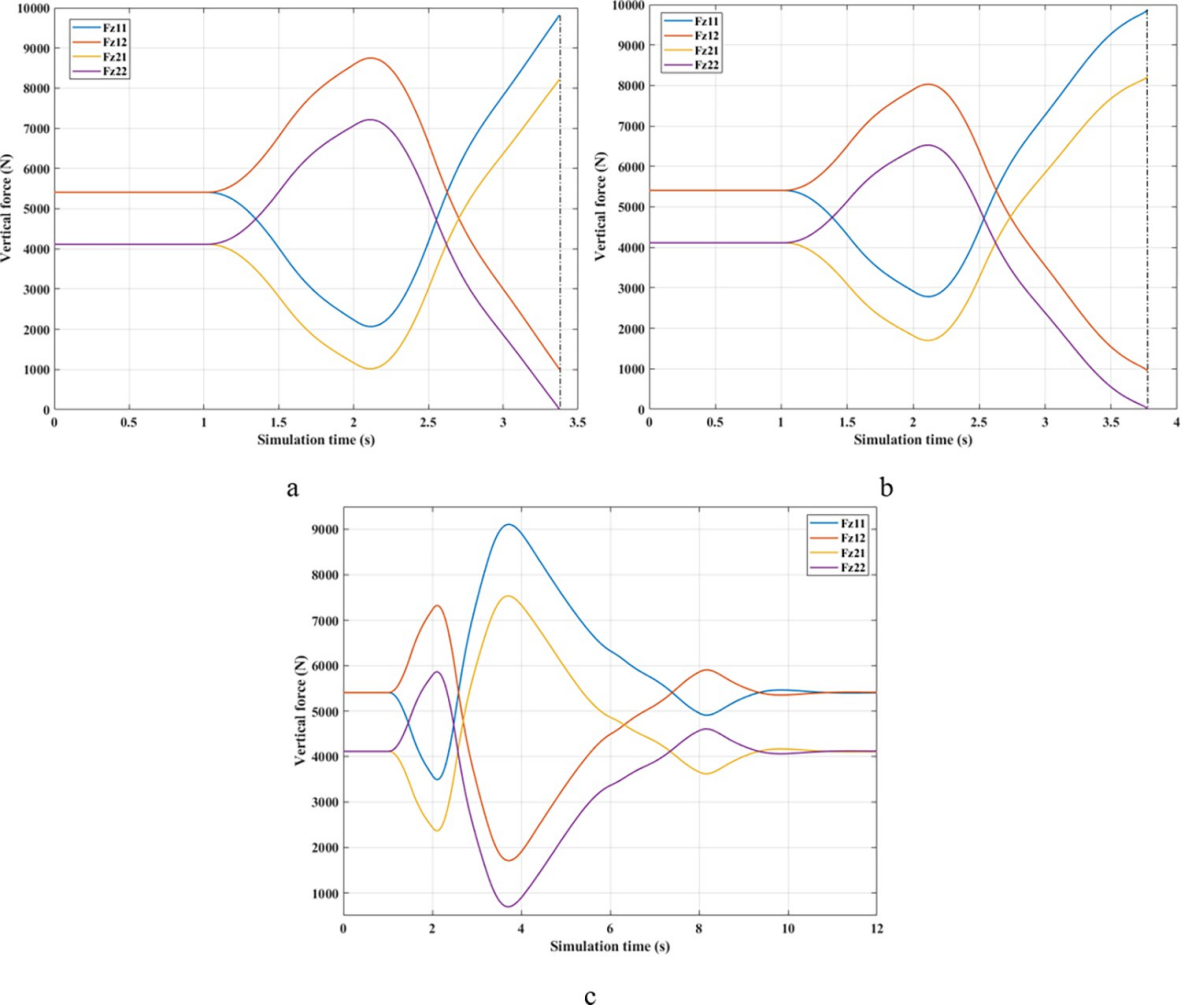
Vertical force (2^st^ case–v_2_). a) None; b) Passive; c) Fuzzy Active.

In the first two situations, wheel dynamics in position (22) rapidly decrease to zero when rollover occurs. According to Fig 21A, the wheel is detached from the road at time t = 3.38 (s) when the car has no stabilizer bars. In the case of vehicles utilizing mechanical anti-roll bars on both the rear and front axles, this occurs after 0.40 (s), i.e., t = 3.78 (s) (Fig 21B). Vehicle stability is only guaranteed once it uses hydraulic stabilizer bars controlled by fuzzy algorithms. When traveling at *v*_*2*_ = 80 (km/h), the stabilizer bars work harder to maintain the interaction between the road surface and wheels. According to the calculation results, the minimum dynamic force value of the right rear wheel (22) is 696.80 (N). This is a small value but still ensures that the car can avoid the phenomenon of rolling over.

**Fig 22:**
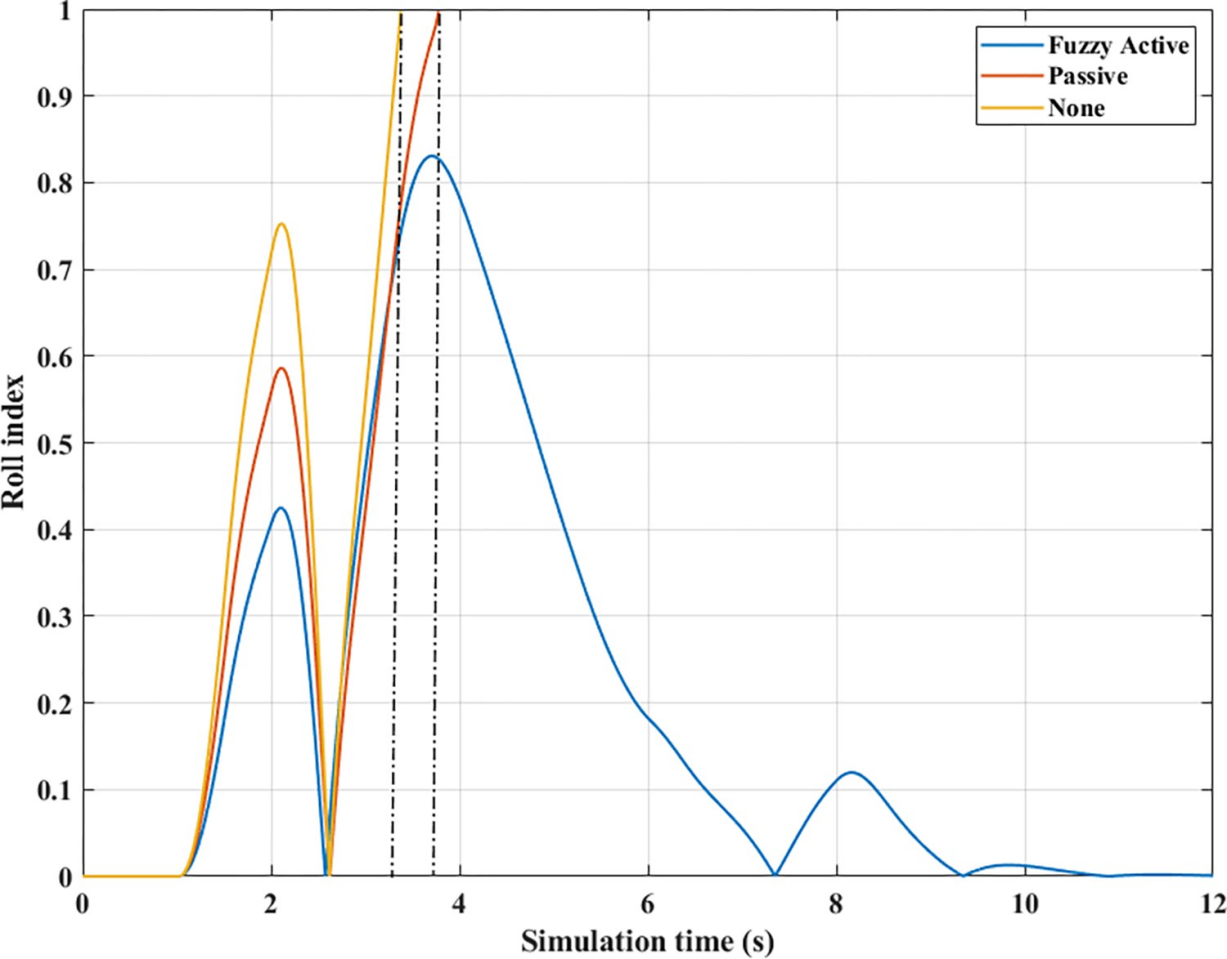
Roll index (2^st^ case–v_2_).

For two situations (None) and (Passive), the rollover index approaches 1, which means the vehicle is completely rolled over. While the maximum roll index of the car when utilizing active stability bars is only 0.83 (Fig 22). However, this is also a dangerous threshold that can easily lead to a rollover if the speed and the steering angle are increased.

**Fig 23:**
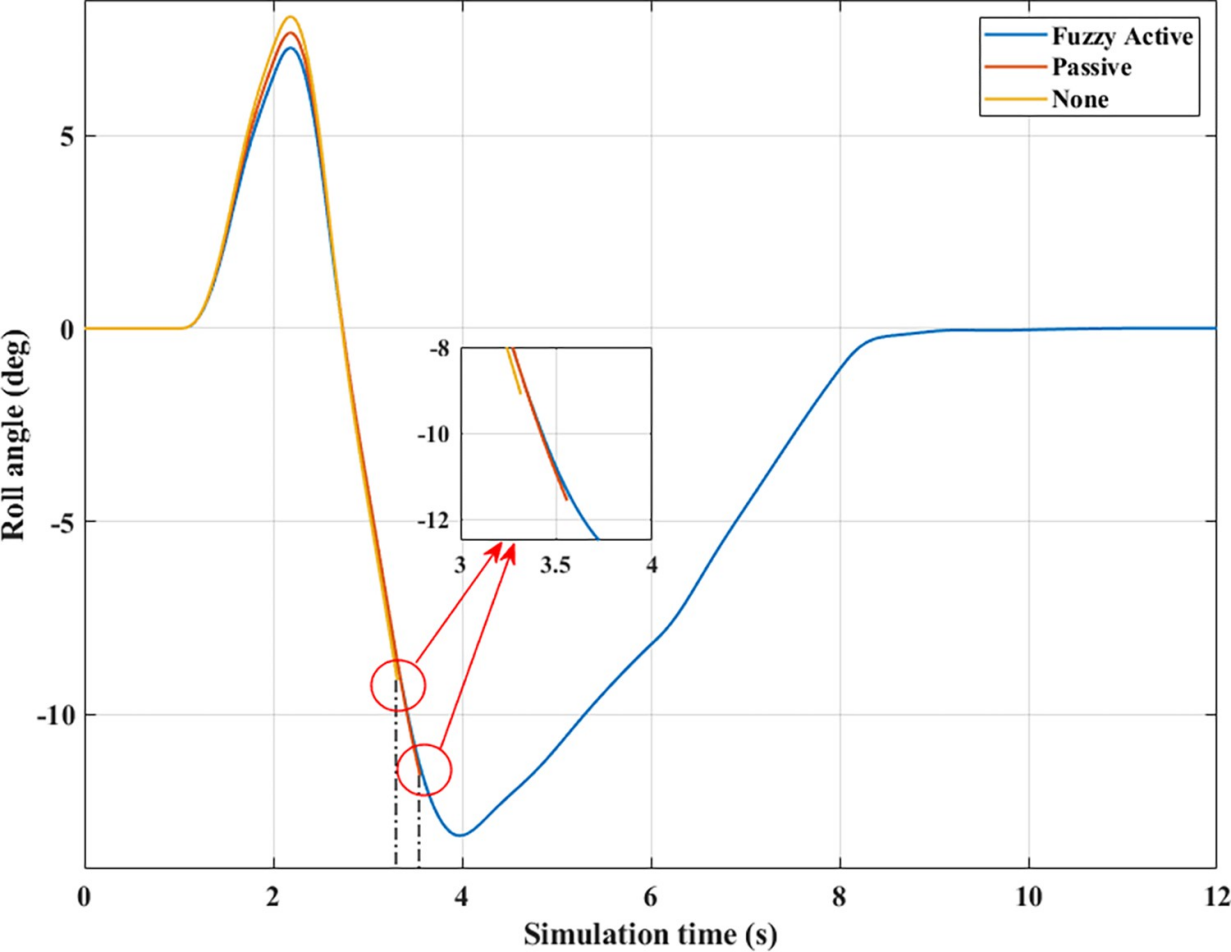
Roll angle (2^st^ case–v_3_).

*v*_*3*_ *= 90 (km/h)*. When the car travels at high speed, *v*_*3*_ = 90 (km/h), roll instability can be described more clearly. According to Fig 23, the rollover occurs earlier for both situations: (None) and (Passive). At time t = 3.31 (s), the car rolls over without stabilizer bars, while this happens at time t = 3.56 (s) when the car has mechanical anti-roll bars. The time to roll over in the condition of traveling with speed *v*_*3*_ is earlier than in the condition of *v*_*2*_, so the peak value of the roll angle is also smaller, only reaching 9.11° and 11.58°, respectively. The car’s roll angle in a situation with active stabilizer bars can be up to 13.13° without rolling. This is an extremely high value and is almost unattainable for cars without an anti-roll bar.

**Fig 24:**
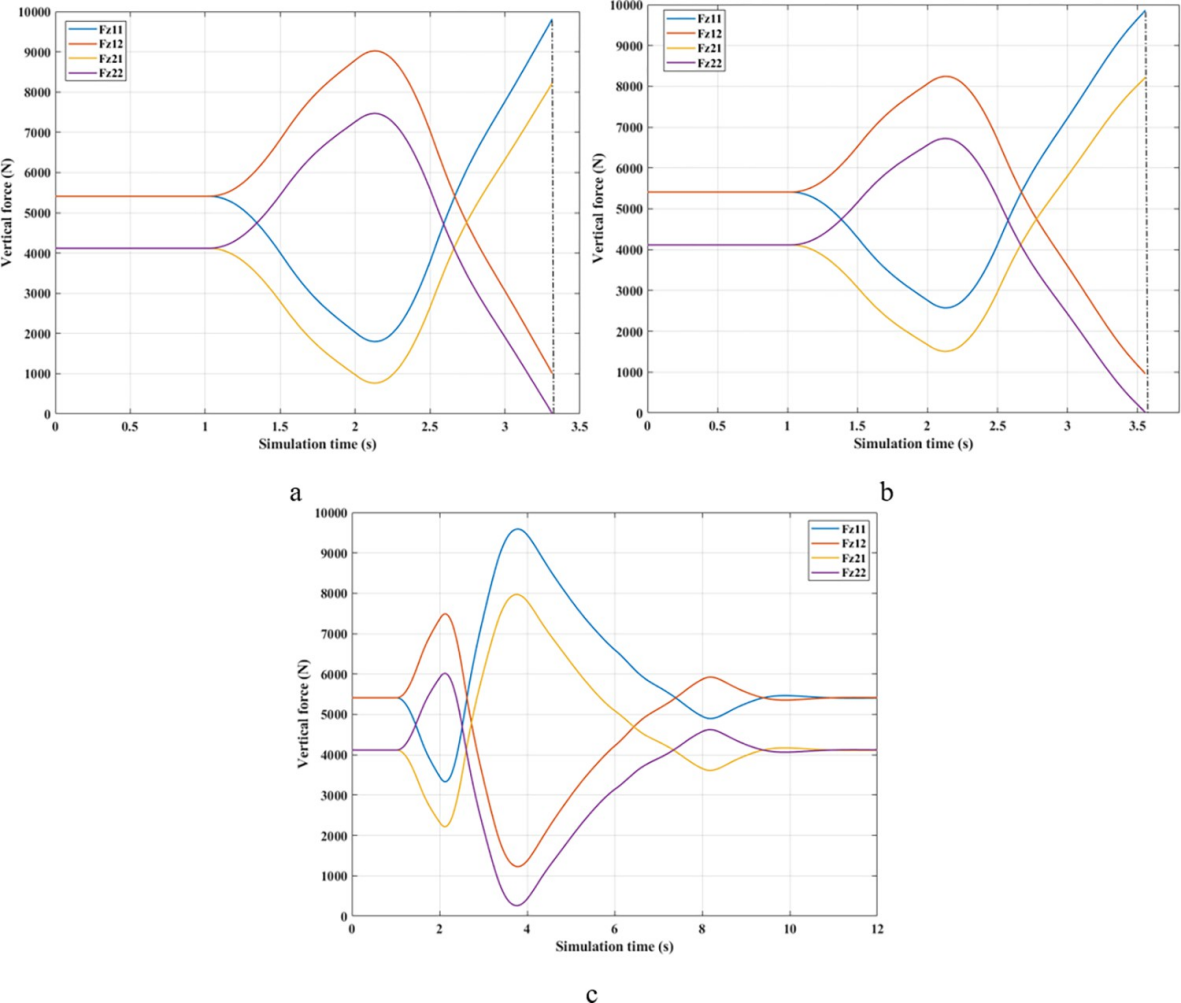
Vertical force (2^st^ case–v_3_). a) None; b) Passive; c) Fuzzy Active.

**Fig 25:**
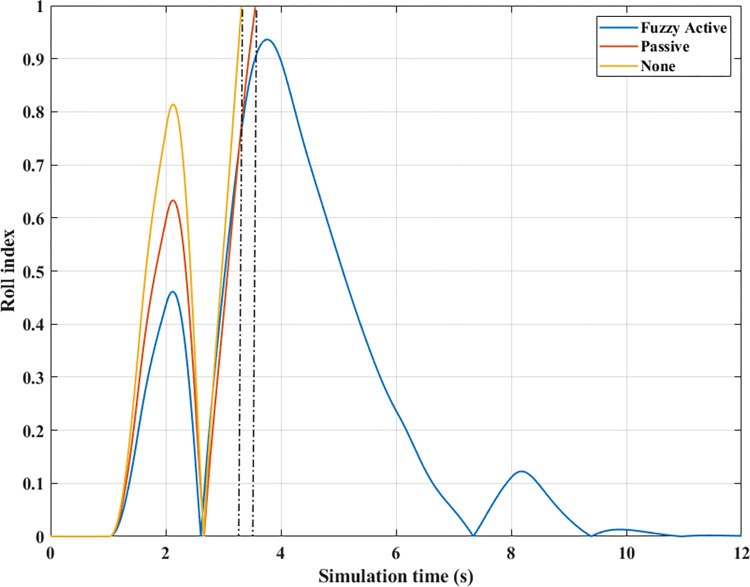
Roll index (2^st^ case–v_3_).

According to Fig 24, the dynamic force at the wheel is declined to zero for two situations (Passive) and (None), while this value still reserves a small amount in the situation (Fuzzy Active) with 262.96 (N). This extremely small number signals that rollover can occur if the driving conditions are more severe, *RI* = 0.93 (Fig 25). Active stabilizer bars can help ensure safety in various oscillation conditions, demonstrated through the interaction between the road surface and the wheel.

#### 3.2.3. Discussion

The findings obtained from calculations and simulations are listed in Tables 3 and 4. Looking at the results more closely, one can see that using stabilizer bars reduce the roll angle’s value. The operating efficiency of the active anti-roll bar is higher than that of the passive anti-roll bar, i.e., the roll angle is reduced more under the same survey conditions. Ensuring the roll angle is stable reduces the load difference between the two sides of the wheel, i.e., more effectively ensuring the interaction between the wheel and the road surface. The overall result is to prevent the rollover that occurs when steering, especially at high speeds and large steering angles.

**Table 3:** Simulation results (1^st^ case).

	Maximum roll angle	Minimum vertical force	Maximum roll index
	v_1_ = 70 (km/h)		
**None**	8.35	715.28	0.83
**Passive**	7.98	1447.33	0.65
**Fuzzy Active**	7.37	2563.85	0.38
	v_2_ = 80 (km/h)		
**None**	9.49	252.43	0.94
**Passive**	9.05	1091.28	0.73
**Fuzzy Active**	8.35	2341.46	0.43
	v_3_ = 90 (km/h)		
**None**	**9.81**	**0**	**1**
**Passive**	10.01	773.06	0.81
**Fuzzy Active**	9.28	2133.11	0.48

**Table 4:** Simulation results (2^nd^ case).

	Maximum roll angle	Minimum vertical force	Maximum roll index
	v_1_ = 70 (km/h)		
**None**	**9.52**	**0**	**1**
**Passive**	10.65	554.61	0.87
**Fuzzy Active**	10.23	1147.25	0.71
	v_2_ = 80 (km/h)		
**None**	**9.29**	**0**	**1**
**Passive**	**11.93**	**0**	**1**
**Fuzzy Active**	11.70	696.80	0.83
	v_3_ = 90 (km/h)		
**None**	**9.11**	**0**	**1**
**Passive**	**11.58**	**0**	**1**
**Fuzzy Active**	13.13	262.96	0.93

Dark gray: Rollover, Light gray: Warning.

Even though cars use mechanical anti-roll bars, rollover still occurs under some driving conditions (case 2: *v*_*2*_ and *v*_*3*_). These problems are overcome by equipping active anti-roll bars directed by an original fuzzy method designed in this work. In the most dangerous case, the vehicle’s roll stability is still guaranteed if it uses active stabilizer bars. According to the results from Table 4, the maximum roll angle at *v*_*3*_ speed can be up to 13.13° while still ensuring the holding of the wheels and the road surface. The rollover occurs entirely for the other two situations (None and Passive).

Compared with the previously applied algorithms, this novel algorithm has many outstanding points, such as ensuring stability while maintaining the comfort and holding of the wheels. However, some disadvantages still exist, such as the object’s convergence and the factors related to motion inertia.

## 4. Conclusion

While steering under the impact of centrifugal force, vehicle rollover instability may occur. The active stabilizer bar has the role of ensuring safety and limiting the rollover phenomenon when the automobile is steering at high speed. In this study, the model of a complex dynamic is used to describe the vehicle rollover oscillations, which include other nonlinear factors. A novel fuzzy method is designed for hydraulic stability bars based on safety and stability criteria. Numerical simulations are performed to help assess the performance of active bars, which takes place in the Simulink environment.

According to research results, the body roll angle increases as the speed and/or the steering angle increase. This reduces the vertical force at the wheel inside the turning arc and eliminates the road surface interaction with the wheel, leading to an increased risk of rolling over, as illustrated by the roll index. When the car uses anti-roll bars, the roll angle and index are reduced, while the interaction between wheels and the road is more ensured.

The simulation results show that the vehicle easily rolls over when the stabilizer bar is unused. Even if the car is equipped with passive anti-roll bars on both rear and front axles, a rollover can still occur if the automobile is steering at high speed. However, this is not the case in situations where the car uses active stability bars instead of conventional mechanical bars. The fuzzy control algorithm provides high efficiency in active bar operation. The automobile’s safety and stability are guaranteed in various complex driving conditions. While the active anti-roll bar and the original fuzzy solution can help limit rollover, this does not mean that they can guarantee complete anti-roll in all cases.

The above results were obtained from simulation without experiment; this is a limitation of the study that needs to be improved in the future. Besides, other issues, such as the environment, disturbances, and uncertain parameters, may affect results. Appropriate solutions are needed to improve these problems.

## Abbreviations

ϕ: Roll angle-rad
ϕ_m_: Motor shaft angle-rad
ψ: Yaw angle-rad
δ: Steering angle-rad
θ: Pitch angle-rad
β: Heading angle-rad
a_y_: Lateral acceleration-ms^-2^
F_ASB_: Active stabilizer bar force-N
F_C_: Damper force-N
F_cex_: Longitudinal centrifugal force-N
Fc_ey_: Lateral centrifugal force-N
F_ims_: Vertical inertia force (sprung mass)-N
F_imu_: Vertical inertia force (unsprung mass)-N
Fix: Longitudinal inertia force-N
F_iy_: Laterall inertia force-N
F_K_: Spring force-N
F_KT_: Spring tire force-N
F_x_: Longitudinal dynamic tire force-N
F_y_: Lateral dynamic tire force-N
F_z_: Vertical dynamic tire force-N
i(t): Control signal-A
M_ϕ_: Roll inertia moment-Nm
M_θ_: Pitch inertia moment-Nm
M_ASB_: Active stabilizer bar moment-Nm
M_z_: Aligning tire moment-Nm
T_r_: Resistance moment-Nm
v_x_: Longitudinal velocity-ms^-1^
v_y_: Lateral velocity-ms^-1^
x_sv_: Servo valve displacement-m
z_s_: Vertical displacement (sprung mass)-m
z_u_: Vertical displacement (unsprung mass)-m
